# NAD^+^/Nrf2 signaling promotes osteogenesis by regulating oxidative level of BMSCs under mechanical stress

**DOI:** 10.1186/s40510-025-00566-2

**Published:** 2025-05-30

**Authors:** Huiying Ren, Jixiao Wang, Jiani Liu, Zijie Zhang, Lingyun Wang, Fulan Wei

**Affiliations:** Department of Orthodontics, School and Hospital of Stomatology, Cheeloo College of Medicine, Shandong University & Shandong Key Laboratory of Oral Tissue Regeneration & Shandong Engineering Research Center of Dental Materials and Oral Tissue Regeneration & Shandong Provincial Clinical Research Center for Oral Diseases, No.44-1 Wenhua Road West, Jinan, 250012 Shandong China

**Keywords:** Mechanical stress, Bone marrow mesenchymal stem cells, Oxidative homeostasis, NAD^+^, Nrf2, Osteogenic commitment

## Abstract

**Background:**

Mechanical stress triggers an increase in cellular reactive oxygen species (ROS), which is associated with the impairment of osteogenesis. During orthodontic treatment, bone marrow mesenchymal stem cells (BMSCs) experience mechanical stress, yet the oxidative profile and redox regulatory mechanisms under such stress, especially involving Nicotinamide adenine dinucleotide (NAD^+^), are not well understood, necessitating further research into their roles in orthodontic therapies.

**Methods:**

The Tension System was established to detect ROS changes in BMSCs under cyclic stretch stress, with H_2_O_2_ simulating uncontrolled ROS. Flow cytometry and fluorescence staining measured ROS, while an NAD^+^/NADH assay kit assessed NAD^+^ levels. qRT-PCR and Western blotting analyzed expression of NAD^+^ synthesis and consume enzymes. Osteogenic potential was evaluated by qRT-PCR, Western blotting, and Alkaline phosphatase (ALP) staining. Loss-of-function and supplementation assays explored role of NAD^+^ in oxidative stress and Nrf2 regulation, with localization assessed by immunofluorescence and Western blotting. In vivo osteogenic effects were confirmed using an orthodontic tooth movement (OTM) model, with osteogenesis assessed by immunohistochemistry and microCT for OTM measurements.

**Results:**

Cyclic stretch stress increased ROS in BMSCs over 24 h and boosted osteogenic differentiation. However, increased ROS from H_2_O_2_ hindered this process. Notably, NAD^+^ levels rose with cyclic stretch, and experiments showed it supported osteogenesis by controlling ROS level in BMSCs. Furthermore, NAD^+^ regulated BMSC ROS via Nrf2 nuclear translocation. Rat models indicated that NMN supplementation enhanced osteogenic and osteoclastic markers and accelerated tooth movement, while FK866 inhibited this effect.

**Conclusions:**

We identified that NAD^+^/Nrf2 signaling regulated oxidative level and thus promoted osteogenic commitment of BMSCs under cyclic stretch stress. Targeting NAD^+^ metabolism or administrating exogenous supplementation to promote bone rebuilding could be a prospective therapy to accelerate OTM.

**Supplementary Information:**

The online version contains supplementary material available at 10.1186/s40510-025-00566-2.

## Introduction

Malocclusion, identified by World Health Organization (WHO) as one of the three major oral diseases, profoundly impacts the dental-maxillofacial functions, facial esthetics and oral health related quality of life (OHRQoL) [1]. Orthodontic treatment, the preferred method for solving malocclusion deformities, is expected to align mal-positioned teeth on the premise of bone homeostasis [2]. Bone homeostasis, comprising bone formation and resorption, is the foundation of bone remodeling and requires multiple factors including normal mechanical loading, proper nutrition, redox balance, etc [3]. In the temporal process of remodeling, bone marrow mesenchymal stem cells (BMSCs) respond to mechanical force at the early stage, playing a central role in osteogenic differentiation and subsequent osteoclast formation [4, 5]. As a consequence, unveiling the homeostatic mechanisms of BMSCs in bone remodeling is crucial for healthy treatment. However, knowledge on redox regulatory mechanism of BMSCs under mechanical stress is not yet fully understood.

Mechanical stress applied to somatic cells can cause fluctuations in the cellular redox environment, in which uncontrolled reactive oxygen species (ROS) can impair DNA, cell structure and physiological functions [6]. Accumulating evidence indicate that dysregulation in ROS level of BMSCs is a crucial event in osteogenic impairment [7, 8]. To avoid oxidative damage, cells exhibit specific metabolic profiles that correspond to their mechanical microenvironment [9]. For instance, selenophosphate synthase and superoxide dismutase (SOD) play pivotal roles in the regulatory mechanisms of redox homeostasis, which help to mitigate oxidative stress [6, 10]. Recently, NAD^+^ was found to be a crucial co-enzyme in skeletal system development [11]. Systemic NAD^+^ deficiency has been implicated in skeletal deformities during development in both humans and mice [12]. Apart from widely used as a cofactor or substrate for biochemical reactions, NAD^+^ has been tightly associated with the progression of oxidative stress-related diseases, including atherosclerosis, arthritis, diabetes and cancer [13, 14]. For example, impairment of salvage pathway decreased NAD^+^ synthesis and exacerbates liver oxidative damage [15]. Meanwhile the supplementation of exogenous Nicotinamide mononucleotide (NMN) has been shown to be protective against the inflammatory and oxidative injuries in the hippocampus region of septic mice through NAD^+^/SIRT1 pathway [16]. However, it remains unclear whether NAD^+^ plays a role in the process of BMSCs adapting to the mechanical environment.

Aiming to address this knowledge gap, the present study seeks to clarify the alterations in ROS level caused by cyclic stretch stress (CSS) and their effects on the osteogenic differentiation potential of BMSCs. We intend to investigate the role and mechanism of NAD^+^ in preserving redox homeostasis in BMSCs under CSS and to ascertain whether exogenous supplementation can accelerate orthodontic tooth movement (OTM) predicated on BMSCs osteogenic activity. The insights gained from this research could pave the way for safer and more effective advancements in orthodontic treatment.

## Methods

### Cell culture and identification of BMSCs

All experiments involving animals in this study were approved by the Medical Ethical Committee of School of Stomatology, Shandong University (No. 20230349). BMSCs were extracted and grown employing methodologies that have been previously outlined [5]. Briefly, mandibles devoid of molars and soft tissues were sourced from four-week-old Wistar rats, which were subsequently treated with 3 mg/mL of collagenase I (Solarbio Science, China) and 4 mg/mL of dispase II (Roche, USA) at a temperature of 37 ℃ for a duration of 2 h. The cells were subsequently harvested and maintained in a primary medium that comprised α-minimal essential medium (α-MEM; BasalMedia, China), 20% fetal bovine serum (FBS; Lonsa Science Srl, Uruguay), and a concentration of 10,000 U/ml penicillin-streptomycin (Biosharp, China). The BMSCs utilized in this research were of the 3rd to 4th passage.

The phenotype of BMSCs was ascertained through the use of flow cytometry. To summarize, ABMMSCs were suspended in phosphate-buffered saline (PBS; BasalMedia, China). Subsequently, antibodies specific to CD44, CD31 (BD; Biosciences), CD90, and CD45 (Elabscience, China) were utilized to identify the phenotype of BMSCs according to the manufacturer’s instructions.

### Osteogenic differentiation and adipogenic differentiation of BMSCs

In summary, BMSCs were grown in an adipogenic-induction medium (AM) that included 0.5 M 3-isobutyl-methylxanthine, 10 µg/mL insulin, 0.2 mM indomethacin, and 1 µM dexamethasone. Following a 21-day period of adipogenic stimulation, the cells were fixed and stained with Oil Red O (Solarbio Science, China) to visualize lipid droplets.

For osteogenic differentiation, BMSCs were cultivated in an osteogenic-induction medium (OM) comprising 50 µg/mL vitamin C, 10 mM β-glycerophosphate, and 10 nM dexamethasone (Sigma-Aldrich, Shanghai, China). After a 7-day osteogenic induction, an ALP staining test was performed to identify calcium deposits using the ALP staining kit (Beyotime Biotechnology, Shanghai, China), following the manufacturer’s instructions. At the conclusion of a 21-day osteogenic induction, the cells were stained with 1% Alizarin Red (pH adjusted to 4.2; Sigma-Aldrich, Shanghai, China) to assess the formation of mineralized nodules.

### Application of mechanical force in vitro

**Fig. 1:**
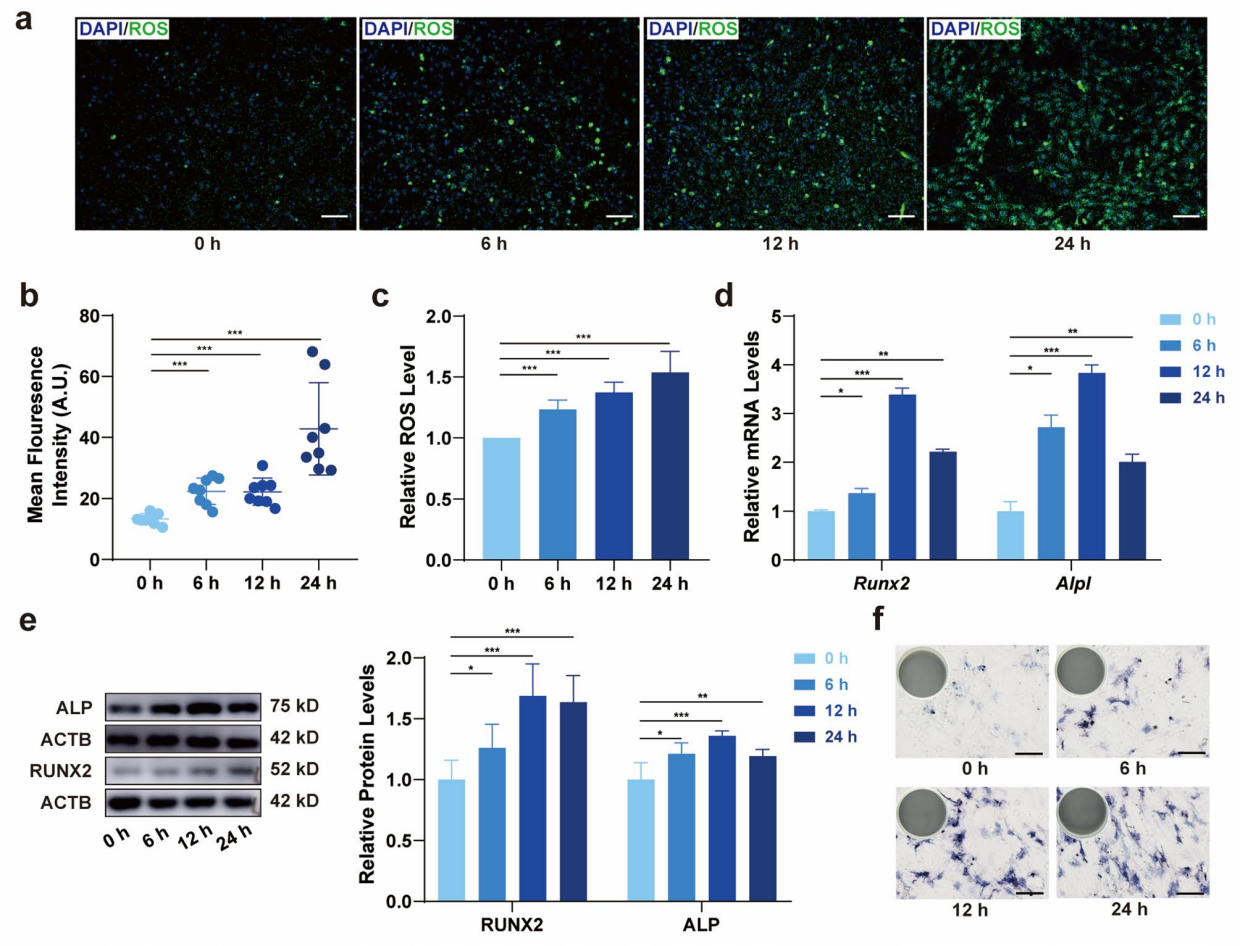
CSS increased ROS level and promoted osteogenic differentiation of BMSCs. a, b The level of ROS in BMSCs after 0, 6, 12, and 24 h of stretching, visualized by fluorescence staining and semi-quantitative analysis. Scale bar, 100 μm. **c** The level of ROS in BMSCs after 0, 6, 12, and 24 h of stretching, visualized by flow cytometry (*N* = 4). **d** The mRNA expression of *Runx2* and *Alpl* in BMSCs during CSS loading. **e** The ratio of the protein levels of RUNX2 and ALP in BMSCs during CSS loading. **f** ALP staining after 0, 6, 12, and 24 h of stretching. Scale bar, 200 μm. * *P*<0.05, ** *P*<0.01, *** *P*<0.001

As distracted in additional file 2 figure S2, BMSCs up to the 4th passage were plated onto 6-well cell culture dishes with collagen I-coating and amino silicone bottoms (Flexcell International, USA) at a concentration of 2.0 × 10^^5^ BMSCs per well (Fig. 1C). The cells were grown under the previously mentioned standard conditions. Once the cells reached approximately 80% confluence, they were subjected to mechanical stretching using the FX-6000 Tension System (Flexcell International, USA) at a 10% stretch and a frequency of 0.5 Hz, according to prior research [5, 18, 19]. The control group was maintained under the same conditions, except for the absence of mechanical stimulation.

### Detection of ROS

The overall ROS levels within the BMSCs were quantified using the Reactive Oxygen Species (ROS) Colorimetric Assay Kit (Elabscience, China) in accordance with the manufacturer’s guidelines. To do this, the BMSCs were treated with a buffer solution that included 10 mM 2’,7’-dichloro-fluorescein diacetate (DCFH-DA) at a temperature of 37 °C for a duration of 20 min. After this incubation, the cells were rinsed with the buffer solution and subsequently analyzed for ROS levels both visually under a confocal laser scanning microscope and through flow cytometry.

### NAD^+^/NADH assay

NAD^+^ and NADH levels were quantified using the NAD^+^/NADH assay kit with WST-8 (Beyotime Biotechnology, Shanghai, China). To begin with, cell lysates were spun down and the supernatant was collected. A portion of this supernatant served as the substrate for measuring the combined NAD^+^ and NADH content. A separate aliquot was heated to 60 ℃ for 30 min to evaluate the NADH levels. As per the protocol, the supernatant was sequentially mixed with acetate dehydrogenase working solution and a chromogenic substrate. After incubation at 37 ℃ in the dark, the absorbance at 450 nm was measured and used for subsequent calculations and analysis. The total NAD^+^ and NADH concentrations, as determined, were normalized to the protein content of the supernatant.

### siRNA transfaction

Either siNC or siNAMPT/siNrf2 (Hanheng Biology, Shanghai, China) was prepared to a concentration of 50 nM using opti-MEM (Gibco, CA, USA) and combined with the lipo2000 (Thermo Fisher Scientific, Massachusetts, USA) working solution. After a 20-minute incubation period, the siRNA-lipo2000 complexes were introduced to the cells and maintained at 37 ℃ with 5% CO_2_ for a duration of 12 h. Following this, the cells were subjected to mechanical force for an additional 12 h.

### RNA extraction and quantitative reverse-transcription polymerase chain reaction (qRT-PCR)

RNA was extracted using RNAiso Plus (Takara, Japan) in accordance with the protocol provided by the manufacturer. For the synthesis of cDNA, the PrimeScript RT Reagent Kit with gDNA Eraser (Takara, Japan) was employed. Thereafter, quantitative real-time PCR (qRT-PCR) was conducted in triplicate using the LightCycler 480 system (Roche Diagnostics, Switzerland) and TB Green Premix Ex Taq II (Takara, Japan). The relative gene expression was determined employing the 2^^−ΔΔCt^ method. A list of all qRT-PCR primer sequences utilized in this research is presented in additional file 1 Table 1.

### Western blotting

BMSCs, rinsed with PBS, were subjected to lysis using a radioimmunoprecipitation assay buffer (Solarbio Science, China) supplemented with 1% phenylmethylsulfonyl fluoride (Solarbio Science, China) for 5 min at 4 °C, followed by a 15-minute incubation to extract the protein lysate. The proteins were then denatured and resolved on a 10% sodium dodecyl sulfate-polyacrylamide gel electrophoresis (SDS-PAGE) gel, and subsequently transferred onto a 0.2-µm polyvinylidene fluoride (PVDF) membrane (Millipore, USA). The membrane was pre-blocked with a solution of 5% non-fat dry milk in Tris-buffered saline with 0.1% Tween-20 (TBS-T) at a pH of 7.2, followed by an overnight incubation at 4 °C with the selected primary antibodies. After three washes with TBS-T, the membrane was incubated for 1 h at room temperature with the chosen horseradish peroxidase (HRP)-conjugated secondary antibodies and then washed again with TBS-T. Protein bands were detected using an Amersham Imager 600 (General Electric, USA) in conjunction with the ECL Western Blotting Substrate (Biosharp, China). The densitometric analysis of the protein bands was performed using ImageJ software (National Institutes of Health, USA). A list of all primary and secondary antibodies used for the western blotting analysis in this study is detailed in additional file 1 Table 2.

After the detection of RUNX2, the immunoblots were stripped off previous antibodies by incubating in stripping buffer (EpiZyme, PS107, Shanghai) for 15 min and used to detect ALP, NAMPT and ACTB, respectively.

### Immunofluorescence

BMSCs, after being fixed with 4% paraformaldehyde, were treated with 0.1% Triton X-100 in PBS for a duration of 20 minutes, followed by a rinse with TBS and a blocking step with PBS containing 5% bovine serum albumin (BSA, Sigma-Aldrich, USA) for 60 minutes. The cells were then incubated with the selected primary antibodies at 4°C throughout the night and subsequently washed three times with PBS. This was followed by a staining process with fluorophore-conjugated secondary antibodies for 1 hour at room temperature and a final staining with 4’,6-diamidino-2-phenylindole (DAPI, Solarbio Science, China) for 5 min. The outcomes were observed using a confocal laser scanning microscope (FV3000, Olympus, Japan).

For the immunofluorescence characterization of murine alveolar bone tissues, the procedure was similar, with the exception that the tissue sections were pre-treated with 0.1% trypsin (Solarbio Science, China) at 37 °C for 20 min to retrieve antigens before blocking. The fluorescence in the stained tissues was examined using a BX51 fluorescence microscope (Olympus, Japan) as well as a confocal laser scanning microscope (FV3000, Olympus, Japan). The densitometric quantification of fluorescence intensity was carried out using ImageJ software (National Institutes of Health, USA).

A list of all primary and secondary antibodies utilized for the immunofluorescence characterizations in this study can be found in additional file 1 Table 2.

### Animal model

The rat OTM model was established based on our prior research [5]. A group of 10 Wistar rats, each approximately six weeks old (Charles River, USA) and weighing around 200 g on average, were housed for a period of 3 days under a 12-hour light/dark cycle, with free access to food and water. In each rat, the left maxillary first molar was tied to the upper incisor on the same side using a nickel-titanium closed-coil spring, which was attached to the teeth with a 0.25 mm stainless steel wire (TOMY, Japan). The wire was fixed with photocurable resin in a groove on the upper incisors. This apparatus exerted a force of about 20 g. The rats were evenly split into two groups labeled A and B, with the dental device left in place for 0 and 7 days, respectively.

An additional 45 rats were divided into three groups labeled C, D, and E, with the dental device maintained as previously described. Group C received regular intraperitoneal injections of 1 mL PBS every 2 days. Group D was given intraperitoneal injections of 100 mg/kg NMN (Beyotime Biotechnology, Shanghai, China) solution in normal saline every 2 days [17]. Group E had FK866, dissolved in DMSO in compliance with the manufacturer’s guidelines, locally injected around the first molar where orthodontic force was applied. During the procedure, the sequence of operations on each rat and the cage positions were randomized.

After an overdose of 3% pentobarbital sodium, the left dentition of each rat, serving as the tooth movement model, and the right dentition, serving as a control, along with their alveolar bone, were collected and fixed in 10% formalin for 24 h. The bone tissue was then demineralized in 14% ethylenediaminetetraacetic acid (EDTA) for a period of 2 months. The paraffin-embedded samples were sectioned into 5-um slices and underwent alcohol gradient dehydration.

### MicroCT scanning

The rat mandibles, after being harvested, were fixed in a 4% paraformaldehyde solution for 24 h and then soaked in a 75% ethanol solution before microCT scanning with a Quantum GX2 scanner (PerkinElmer, Shelton, Connecticut, USA). The scan was conducted using standard protocols at 90 kV, 88 µA, with a voxel size of 72 μm. The resulting data was analyzed with CTAn software (Skyscan, Bruker, Billerica, MA, USA) to determine bone volume fraction (BV/TV), trabecular number (Tb.N), and trabecular thickness (Tb.Th) on the tension side of the first molar.

### H&E staining

Sections of murine alveolar bone tissue were prepared according to the standard protocols. Briefly, the tissue sections were deparaffinized in xylene and rehydrated through a graded ethanol series to distilled water. Subsequently, the sections were immersed in hematoxylin solution (Solarbio Science, China) for 5 min to stain the nuclei. After thorough rinsing with running tap water, the sections were differentiated in 1% acid alcohol for 10 s to enhance nuclear contrast, followed by a brief wash in running tap water. The sections were then counterstained with eosin solution (Solarbio Science, China) for 2 min to highlight the cytoplasm and extracellular matrix components. Following a final rinse in distilled water, the sections were dehydrated through a graded ethanol series, cleared in xylene, and mounted with a coverslip using neutral resin. The stained sections were examined under a BX51 light microscope (Olympus, Japan) and photomicrographs were captured for further analysis.

### Immunohistochemistry

The sections of murine alveolar bone tissue were prepared through the antigen retrieval step outlined previously and subsequently treated with 3% hydrogen peroxide at 37 °C for a period of 20 min. After being blocked with BSA, the samples were incubated with the primary antibodies, followed by the HRP-conjugated secondary antibodies, adhering to the procedures previously mentioned. The tissue sections were then stained with diaminobenzidine and hematoxylin (Solarbio Science, China) and examined using a BX51 fluorescence microscope (Olympus, Japan). The densitometric quantification of positively stained cells was performed using ImageJ software (National Institutes of Health, USA). A compilation of all primary and secondary antibodies employed for immunohistochemical staining in this study is presented in additional file 1 Table 2.

### Statistical analysis

Data from all experiments that exhibited a normal distribution were presented as the mean ± standard deviation, derived from a minimum of three separate trials, and were processed using GraphPad Prism 9.0 software (GraphPad Software Inc., San Diego, USA). Statistical comparisons between pairs of groups were made using Student’s *t*-test. A *p*-value of less than 0.05 was considered statistically significant. The error bars depicted in the figures represent the standard deviation from at least three replicates, unless otherwise specified.

## Results

### CSS increased ROS level and promoted osteogenic differentiation of BMSCs

BMSCs were successfully isolated from mandible of Wistar rats. The osteogenic and adipogenic potentials were confirmed by Alizarin Red S staining for calcium nodules and Oil Red O staining for lipid droplets (Additional file 2: Fig. S1a). Flow cytometry analysis revealed that the cells were positive for CD44 and CD90, and negative for CD31 and CD45 (Additional file 2: Fig. S1b). To investigate the oxidative state of BMSCs, we utilized the Tension System, as depicted in Figure S2. In our in vitro experiments, BMSCs were subjected to CSS for 6, 12, and 24 h, mimicking the mechanical forces encountered in vivo. The ROS levels showed a time-dependent increase, with the highest levels observed after 24 h of stretching (Fig. 1a-c).

Subsequently, we evaluated the impact of mechanical stimuli on the osteogenic differentiation of BMSCs. Considering the potential for post-transcriptional regulation, we examined both the transcriptional and translational levels of genes under CSS. The expression of osteogenesis-related genes (*Alpl* and *Runx2*), began to rise at 6 h, peaked at 12 h and slightly decrease at 24 h (Fig. 1d). Concurrently, the protein levels of ALP and RUNX2 were also significantly increased in response to mechanical force (Fig. 1e). Furthermore, the ALP enzyme activity within the cells was substantially elevated and remained stable after 12 h of cyclic tension (Fig. 1f).

These findings collectively demonstrate that the oxidative state of BMSCs is enhanced by CSS and that their osteogenic differentiation is promoted under CSS.

### Elevated ROS induced by H_2_O_2_ impaired osteogenic differentiation of BMSCs under CSS

**Fig. 2:**
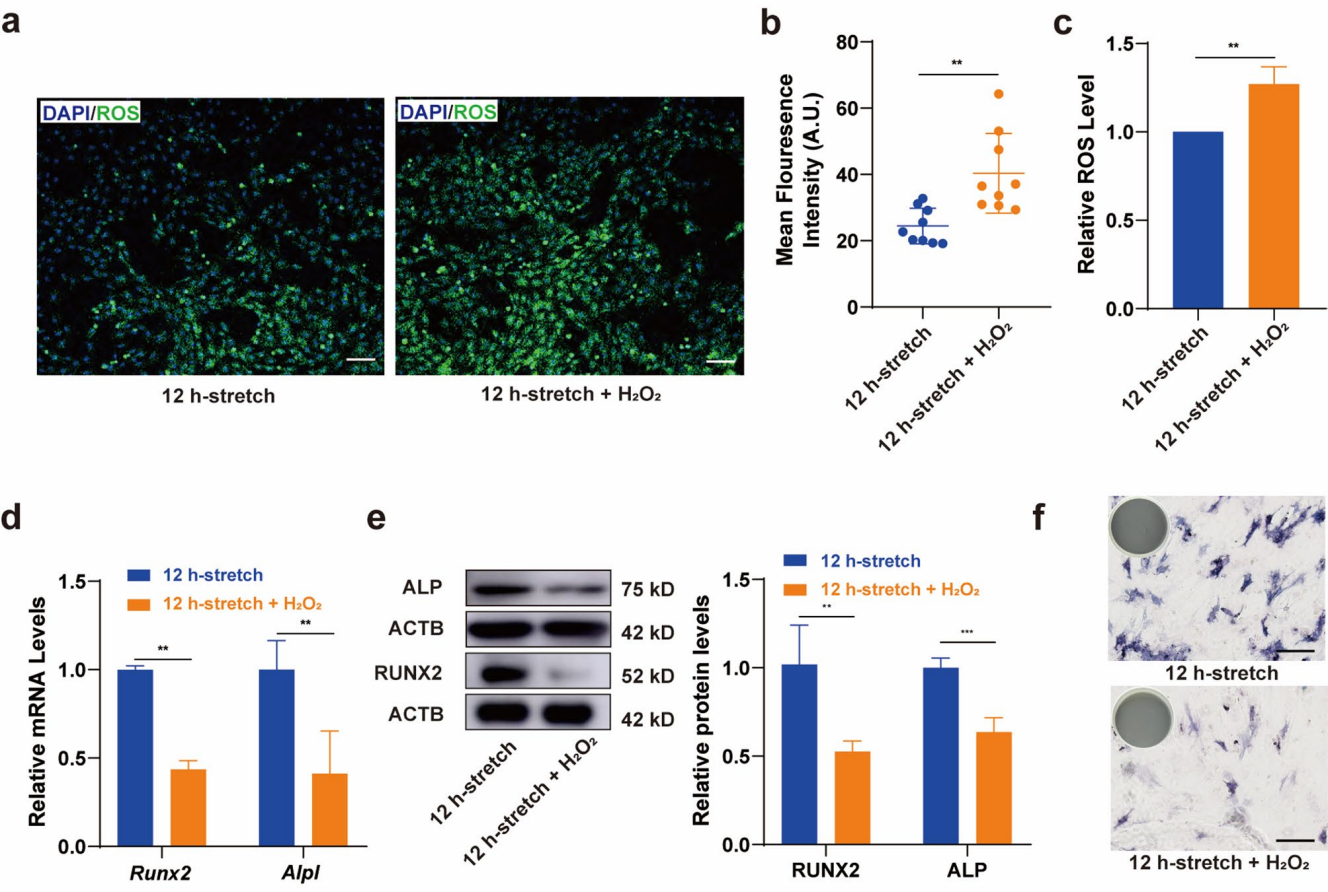
Elevated ROS induced by H_2_O_2_ impaired osteogenic differentiation of BMSCs under CSS. To investigate the impact of ROS on the osteogenic activity of BMSCs under CSS, exogenous ROS were introduced by H_2_O_2_ (200 µM). **a b** The level of ROS in BMSCs after 12 h of stretching with or without 1 mM H_2_O_2_ treatment, visualized by fluorescence staining and semi-quantitative analysis. Scale bar, 100 μm. **c** The level of ROS in BMSCs after 12 h of stretching with or without H_2_O_2_ treatment, visualized by flow cytometry (*N* = 4). **d** The mRNA expression of *Runx2* and *Alpl* in BMSCs after 12 h of stretching with or without H_2_O_2_ treatment. **e** The ratio of the protein levels of RUNX2 and ALP in BMSCs after 12 h of stretching with or without H_2_O_2_ treatment. **f** ALP staining in BMSCs after 12 h of stretching with or without H_2_O_2_ treatment. Scale bar, 200 μm. * *P*<0.05, ** *P*<0.01, *** *P*<0.001

To explore the effects of increased exogenous ROS on osteogenic differentiation in BMSCs, we treated cells that had undergone 12 h of CSS with 200 µM H_2_O_2_. The ROS levels in H_2_O_2_-treated cells were found to be higher than in the cells subjected only to CSS (Fig. 2a-c). Further analysis of the transcriptional and translational profiles of ALP and RUNX2 revealed a reduction in osteogenic differentiation in BMSCs under H_2_O_2_-induced ROS elevation. Additionally, the ALP enzyme activity was also observed to decrease in the H_2_O_2_-treated group. Collectively, these results confirm that oxidative stress negatively affects the osteogenic differentiation of BMSCs under mechanical force.

### NAD^+^ regulated ROS levels and promoted osteogenic differentiation of BMSCs under CSS

**Fig. 3:**
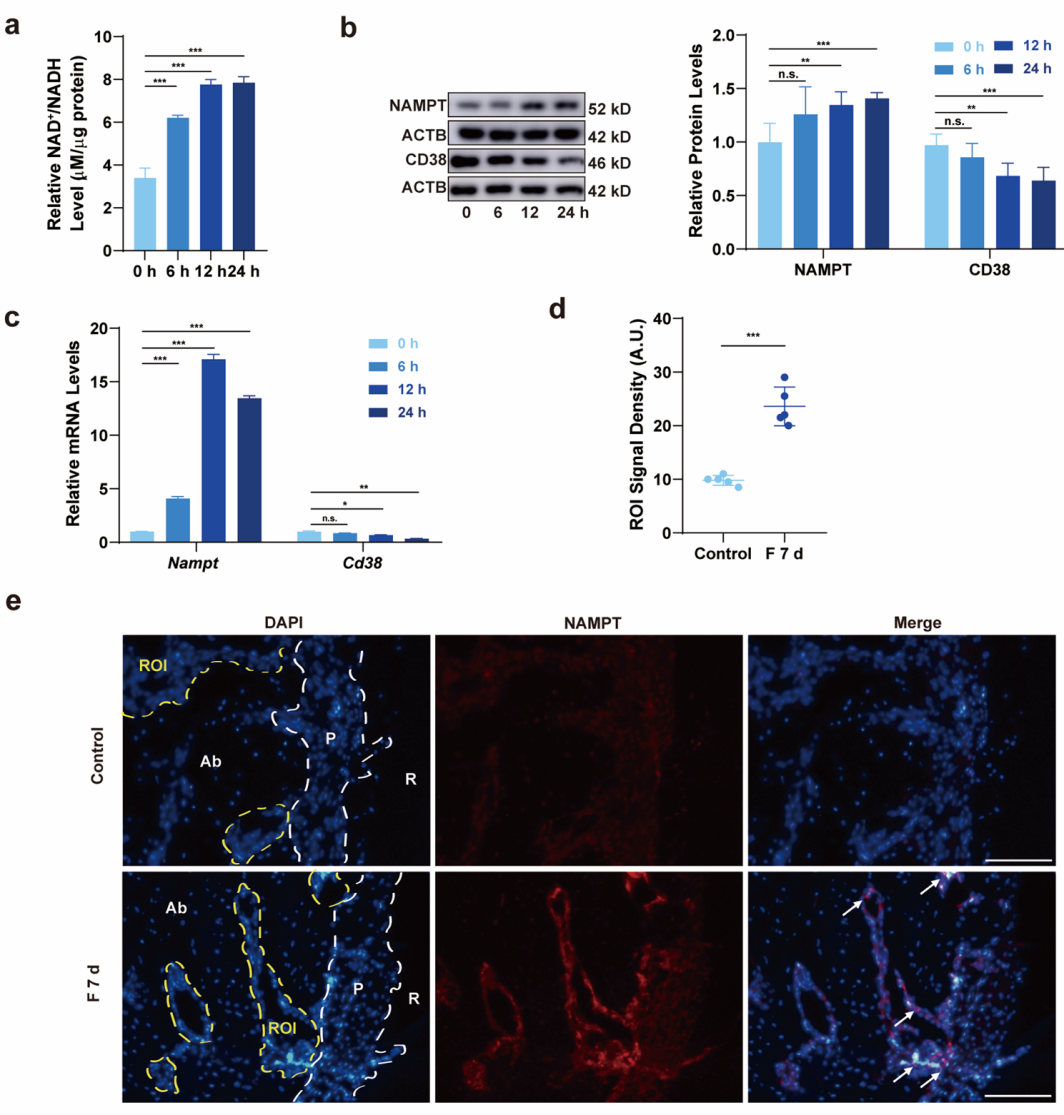
NAD^**+**^ synthesis increased in BMSCs under CSS and OTM. a The level of NAD^+^/NADH produced by BMSCs during 0, 6, 12, and 24 h of stretching. **b** The ratio of the protein levels of NAMPT and CD38 in BMSCs during CSS loading. **c** The mRNA expression of *Nampt* and *Cd38* in BMSCs during CSS loading. **d e** Immunofluorescence and semi-quantitative analysis of NAMPT expression in the bone marrow cavities during mechanical force application. Scale bar, 40 μm

To evaluate the role of NAD^+^ in mechanical loading, we first measured the total NAD^+^ production using the NAD^+^/NADH assay kit (Beyotime Biotechnology, Shanghai, China). Additionally, we examined the mRNA and protein levels of Nicotinamide phosphoribosyltransferase (NAMPT), a key enzyme in the NAD^+^ synthesis salvage pathway [12], and Cyclic ADP-ribose hydrolase (CD38), an enzyme that consumes NAD^+^. The qRT-PCR and Western blotting analyses showed an upregulation of NAMPT and a downregulation of CD38 expression (Fig. 3c d), which aligns with the observed increase in total NAD^+^ production (Fig. 3a). These findings indicate that an endogenous increase in NAD^+^ is required for the osteogenic differentiation of BMSCs under mechanical loading.

To verify the in vivo change of NAD^+^ synthesis in orthodontic remodeling tissue, orthodontic tooth movement (OTM) models were established (Additional file 2: Fig. S3a b). As the difficulty in precisely isolate bone marrow cavity from periodontal ligament, vessels and nerves, NAD^+^ metabolism was characterized by expression of NAMPT. Compared to control group, NAMPT expression at 7 day of OTM was significantly upregulated (Fig. 3d e), consistent with the in vitro findings.

**Fig. 4:**
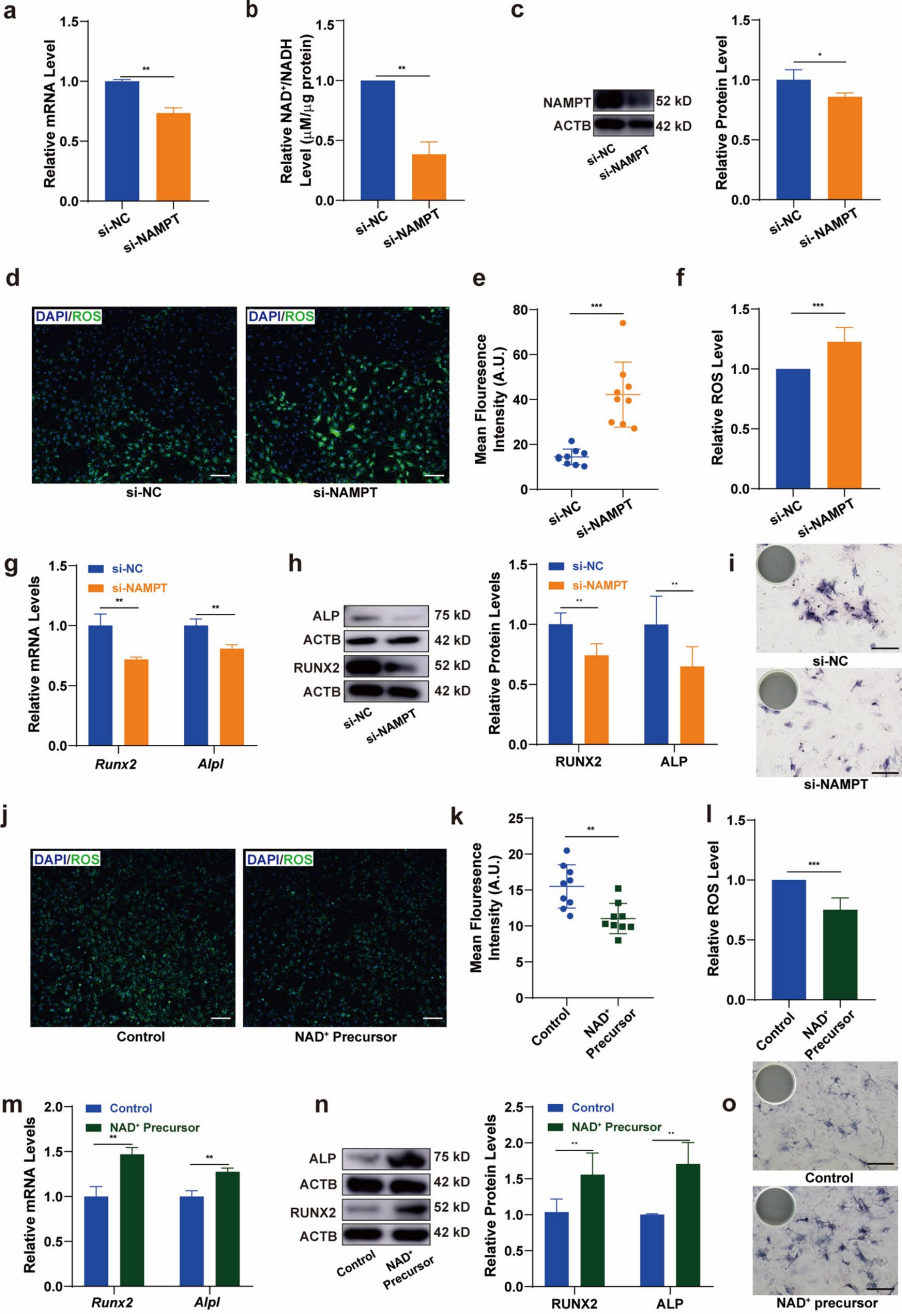
NAD^**+**^ regulated ROS levels and promoted osteogenic differentiation of BMSCs under CSS. To evaluate the impact of NAD^+^ on the oxidative levels and osteogenic differentiation of BMSCs, siRNA was used to knock down NAMPT, a key enzyme for NAD^+^ synthesis, or to supplement NMN, a precursor for NAD^+^ synthesis. **a b c** knockdown efficiency of NAMPT and effect on NAD^+^/NADH production. **d e** The level of ROS in BMSCs after 12 h of stretching with or without NAMPT knocked down, visualized by fluorescence staining and semi-quantitative analysis. Scale bar, 100 μm. **f** The level of ROS in BMSCs after 12 h of stretching with or without NAMPT knocked down, visualized by flow cytometry (*N* = 6). **g** The mRNA expression of *Runx2* and *Alpl* in BMSCs after 12 h of stretching with or without NAMPT knocked down. **h** The ratio of the protein levels of RUNX2 and ALP in BMSCs after 12 h of stretching with or without NAMPT knocked down. **i** ALP staining in BMSCs after 12 h of stretching with or without NAMPT knocked down. Scale bar, 200 μm. **j k** The level of ROS in BMSCs after 12 h of stretching with or without NMN supplement, visualized by fluorescence staining and semi-quantitative analysis. Scale bar, 100 μm. **l** The level of ROS in BMSCs after 12 h of stretching with or without NMN supplement, visualized by flow cytometry (*N* = 6). **m** The mRNA expression of *Runx2* and *Alpl* in BMSCs after 12 h of stretching with or without NMN supplement. **n** The ratio of the protein levels of RUNX2 and ALP in BMSCs after 12 h of stretching with or without NMN supplement. **o** ALP staining in BMSCs after 12 h of stretching with or without NMN supplement. Scale bar, 200 μm. * *P*<0.05, ** *P*<0.01, *** *P*<0.001

To examine the role of NAD^+^ in the oxidative regulation and osteogenic differentiation of BMSCs induced by CSS, we employed small interfering RNA (siRNA) to knock down the expression of NAMPT, thereby reducing NAD^+^ production, and supplemented with NAD^+^ precursor NMN, thereby increase NAD^+^ production. The establishment of the model with low NAD^+^ expression was confirmed through analyses using qRT-PCR, Western blotting, and NAD^+^/NADH Content Assay Kit (Fig. 4a-c). Fluorescence imaging and flow cytometry data revealed an elevated ROS level in the NAD^+^-depleted group, after 12 h of CSS, as shown in Fig. 4d-f. While supplement of NAD^+^ precursor decreased ROS level of BMSCs after 12 h of CSS (Fig. 4j-l). This suggests that NAD^+^ plays a role in regulating the oxidative status of BMSCs. Additionally, the osteogenic differentiation was compromised in the si-NAMPT group (Fig. 4g h), and promoted in the NAD^+^ precursor-added group (Fig. 4m n), as evidenced by the reduced expression of RUNX2 and ALP, determined through qRT-PCR and Western blotting analyses. Concurrently, the ALP enzyme activity was also found to be decreased in the si-NAMPT group and increased in the NAD^+^ precursor-added group (Fig. 4i o).

These results demonstrate that endogenous NAD^+^ controls ROS level of force-induced BMSCs and promotes osteogenic differentiation.

### NAD^+^ regulated ROS level of BMSCs under CSS through Nrf2 nuclear localization

**Fig. 5:**
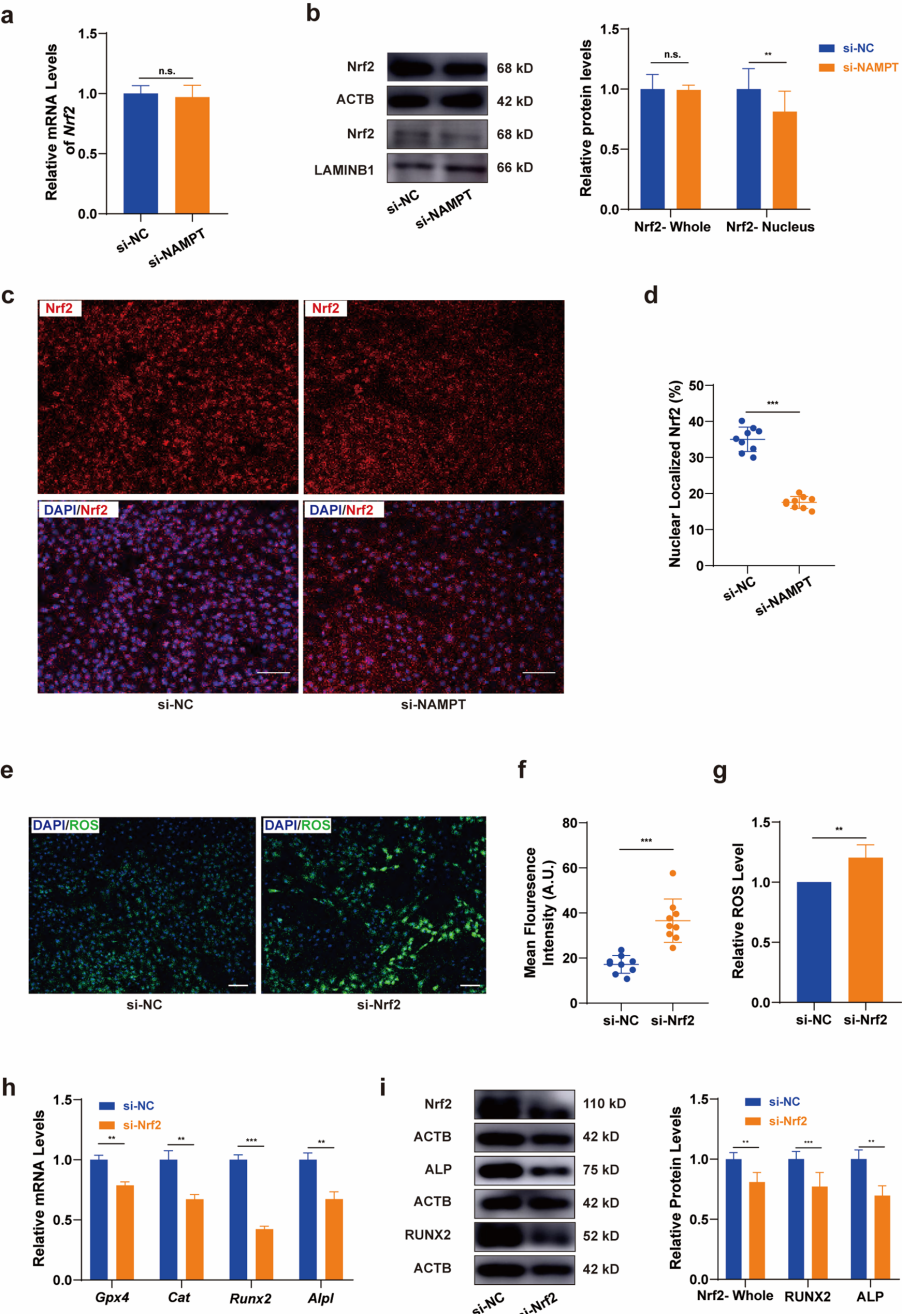
NAD^+^ regulated ROS levels of BMSCs under CSS through Nrf2 nuclear localization. a The mRNA expression of *Nfe2l2* in BMSCs after NAMPT knocked down. **b** The ratio of the protein level and nuclear localization of Nrf2 in BMSCs. **c d** Immunofluorescence and semi-quantitative analysis of Nrf2 nuclear localization in the si-NC and si-NAMPT group after stretching for 12 h. Scale bar, 100 μm. **e f** The level of ROS in BMSCs after 12 h of stretching with or without Nrf2 knocked down, visualized by fluorescence staining and semi-quantitative analysis. Scale bar, 100 μm. **g** The level of ROS in BMSCs after 12 h of stretching with or without Nrf2 knocked down, visualized by flow cytometry (*N* = 6). **h** The mRNA expression of antioxidative gene *Gpx4* and *Cat*, osteogenic marker *Runx2* and *Alpl*, in BMSCs after 12 h of stretching with or without Nrf2 knocked down. **i** The ratio of the protein levels of RUNX2 and ALP in BMSCs after 12 h of stretching with or without Nrf2 knocked down. * *P*<0.05, ** *P*<0.01, *** *P*<0.001

Recognizing Nrf2 as a key transcription factor in maintaining and adapting to intracellular redox homeostasis, we sought to investigate the influence of NAD^+^ on Nrf2 activation and its regulatory mechanisms. To this end, we confirmed the *Nfe2l2* expression on transcriptional level and assessed the relative content of Nrf2 in both whole-cell and nuclear protein extracts of BMSCs treated with siNC (Negative Control) and siNAMPT under 12 h of CSS. As shown in Fig. 5a and b, the downregulation of NAD^+^ did not affect the transcriptional activity or alter the whole-cell protein levels of Nrf2. However, a significant reduction in the nuclear content of Nrf2 was observed in siNAMPT treated BMSCs compared to the siNC group. This indicates that under low NAD^+^ conditions, the nuclear localization of Nrf2 was impeded. Immunofluorescence staining (Fig. 5c) and quantification of nuclear localized Nrf2 (Fig. 5d) further confirmed this result.

To examine the hypothesis that NAD^+^ regulates ROS level and force-induced osteogenic differentiation of BMSCs through Nrf2 nuclear localization, we established a Nrf2 low-expression model. After 12 h of CSS, the group with low Nrf2 expression exhibited a higher ROS content, as evidenced by fluorescence imaging and flow cytometry (Fig. 5e-g). Additionally, the expression of antioxidant genes (*Gpx4* and *Cat*) and osteogenic markers was suppressed(Fig. 5h i). These confirm the hypothesis that Nrf2 and its downstream antioxidant genes participate in NAD^+^-regulated ROS control and BMSCs osteogenic differentiation under CSS.

### NAD^+^ precursor promoted bone remodeling and accelerated OTM, while NAD^+^ inhibitor compromised bone remodeling and inhibited OTM

**Fig. 6:**
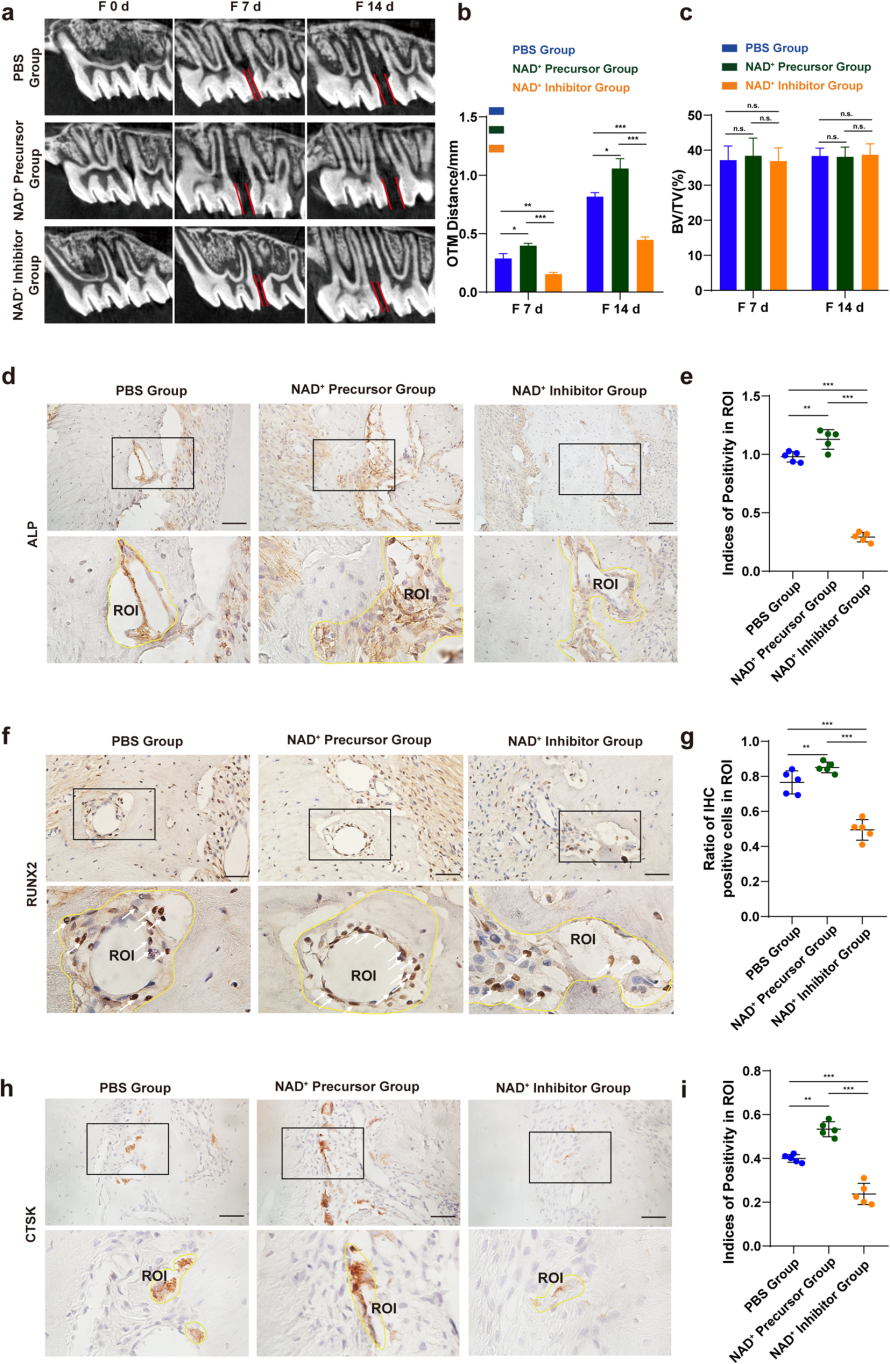
NAD^+^ precursor promoted osteogenesis and accelerated OTM, while NAD^+^ inhibitor compromised osteogenesis and inhibited OTM. To evaluate the impact of NAD^+^ on osteogenesis of alveolar bone and tooth movement under orthodontic force, NAD^+^ precursor NMN and inhibitor FK866 was used in OTM rat models. **a b** Micro-computed tomography of OTM for 0, 7, and 14 days (*N* = 3). **c** Quantification of BV/TV of mechanically stimulated models after injection of PBS, NMN, and FK866 on 7 and 14 days. (*N* = 3). **d e** Immunohistochemistry (IHC) and semi-quantitative analysis of ALP expression in the bone marrow cavities after injection of PBS, NMN, and FK866 on 7 days. Scale bar, 40 μm. **f g** IHC and semi-quantitative analysis of RUNX2 expression in the bone marrow cavities after injection of PBS, NMN, and FK866. Scale bar, 40 μm. **h i** IHC and semi-quantitative analysis of CTSK expression in the bone marrow cavities after injection of PBS, NMN, and FK866. Scale bar, 40 μm. * *P*<0.05, ** *P*<0.01, *** *P*<0.001

To verify the impact of NAD^+^ on osteogenic activity during orthodontic tooth movement in rats, the established OTM rat model was randomly divided into groups and treated with PBS, NMN, and FK866 at a two-day frequency (Additional file 2: Fig. S3a). NMN, a precursor in the NAD^+^ salvage synthesis pathway, can elevate intracellular NAD^+^ levels in vivo [20], while FK866, as a chemical inhibitor of NAMPT, significantly reduces NAD^+^ synthesis [14]. MicroCT scans were used to obtain images of the first molar and surrounding bone tissue on days 7 and 14. It was found that compared to the control group, supplementation with NMN significantly accelerated the rate of tooth movement, while the use of an NAD^+^ synthesis inhibitor in the treatment group resulted in suppressed tooth movement (Fig. 6a b). However, microCT scan and quantification of BV/TV, Tb. N and Tb. Th shown no significant difference among groups of PBS group, NAD^+^ precursor group and NAD^+^ inhibitor group on 7 or 14 days (Fig. 6c and Additional file 2: Fig. S3c d). This might be because mineralized bone tissue that can be detected by X-ray had not yet formed.

Since accelerated OTM can sometimes increase the risk of root resorption, H&E staining was performed to observe and evaluate the root intigrity. No significant root resorption was detected at either 7 or 14 days. Figure. S3 e shows the H&E staining results of the compressed side of the tooth root and periodontal tissues. On the seventh and the fourteenth day of the aforementioned treatment, the alveolar bone tissue was prepared into paraffin sections to observe the osteogenic differentiation. Immunohistochemical results showed that compared with the control group, the expression of ALP on the cell membrane surface and RUNX2 within the cell nucleus increased in the NMN group (Fig. 6d e and Additional file 2: Fig. S4a b), while FK866 significantly inhibited the expression of ALP and RUNX2 (Fig. 6f g and Additional file 2: Fig. S4c d). As to osteoclastic process on alveolar bone, CTSK expression was observed increased in the NMN group and decreased in the FK866 group (Fig. 6h i and Additional file 2: Fig. S4e f).

## Discussion

BMSCs, residing in the bone marrow close to the bone surface, encounter a variety of external biophysical cues, including shear stress [21], hydrostatic pressure, substrate strain [22], and etc. CSS activates osteogenic differentiation of BMSCs, and on the other hand, is an environment disturbance that loads stress on them [23]. Once being disturbed, stem cells activate inherent self-regulatory mechanisms to maintain homeostasis, which is the foundation for adapting to environmental changes [9]. Most current research focuses on exploring the mechanisms by which cells sense and transmit mechanical signals and shift to osteogenic differentiation [4, 24, 25], yet the intrinsic processes by which cells respond to mechanical disturbances and maintain homeostasis remain uncharted. Our study primarily focuses on the changes in oxidative balance and intrinsic regulatory mechanisms of BMSCs under mechanical stimulation, providing a molecular basis for enhancing the mechanical responsiveness and sustained osteogenic capacity of BMSCs.

Our results showed that intracellular ROS levels in BMSCs gradually increased with the duration of mechanical stimulation (Fig. 1A-C). As demonstrated in previous studies, including our own work, osteogenic differentiation of BMSCs is accompanied by a significant increase of oxidative phosphorylation (OXPHOS) [18], which occurs with promoted mitochondrial functions [26]. Along with the improvement of mitochondrial respiratory function, the electron leakage on the inner mitochondrial membrane also increases correspondingly, forming ROS [27]. Therefore, we speculated that the increased ROS level during CSS loading might be a response to mechanical signals and subsequent metabolic reprogramming. We also found an early upregulation of osteogenic markers in BMSCs under CSS (Fig. 1D-F), consistent with the aforementioned conclusions [5]. However, in bone homeostasis, ROS accumulation is typically linked to tissue damage and compromised ossification in aging, inflammation, and pathological states [28, 29]. Therefore, in the subsequent experiments, we induced an increase in ROS levels of BMSCs using H_2_O_2_ (200 µM) [30] and found that exogenous ROS elevation impaired osteogenic process under 12 h CSS (Fig. 2), which aligned with evidences of oxidative stress hindering cell functions. The above results demonstrated an intriguing dichotomy in the role of ROS in osteogenic differentiation under mechanical stress versus exogenous ROS induction. Recent years, the impact of oxidants on the organism is not monolithic; in different physiological or pathological contexts, ROS exhibit roles of “distress” and “eustress“ [31, 32]. Physiological levels of ROS are necessary for cell differentiation and functional regulation [33, 34]. For example, in fracture healing process, ROS serves as a mediator of early cellular mobilization [35]; in metabolic-related disease, ROS functions as a metabolic signal that regulates the balance between bone formation and resorption [36]. On the other hand, evidence suggest that accumulation of ROS under mechanical stress do harm to stem cells and impair differentiation capability [37]. In osteoarthritis, excessive ROS induced from excessive mechanical load is a contributing factor to cartilage degeneration [38]. This discrepancy suggests the presence of a regulatory mechanism that modulates ROS homeostasis in a manner that supports osteogenic activity. In our study, the CD44 receptors expressed on the surface of BMSCs may play a role in the osteogenic differentiation regulated by physiological levels of ROS. ROS can alter the composition and structure of the extracellular matrix, affecting the interaction of CD44 with hyaluronic acid, and participate in cell adhesion and migration, thereby influencing the cells’ ability to adhere and migrate [39]. However, long-term stimulation of CSS on BMSCs exhibited depressed osteogenesis in vitro. This may be due to stress and impairment caused by prolonged mechanical stimulation. According to previous studies, long-term mechanical stimulation (such as continuous cyclic tensile stress for 21 days) can lead to cell apoptosis and impair osteogenesis [40]. However, in other research, the application of intermittent cyclic tensile stress to BMSCs over several consecutive days has been shown to promote osteogenic differentiation of BMSCs [26]. This suggests that the form of mechanical force applied to cells can influence their response to prolonged mechanical stimulation. In our further study, we will focus on long-term effect of mechanical stress on BMSCs or other periodontal cells.

Intracellular ROS must be tightly regulated for proper redox balance within BMSCs to avoid excessive accumulation [32]. However, the intrinsic mechanisms by which cells regulate their redox balance under mechanical stress remain unclear. NAD^+^, serving as the cellular hydrogen carrier for redox reactions, is recognized for its involvement in redox processes in recent years [20]. A steady supply of NAD^+^ is essential during osteogenesis [12]. Upregulation of NAD^+^ was reported to reverse cellular senescence and tissue damage caused by oxidative stress [13]. To evaluate the impact of NAD^+^ on the oxidative levels and osteogenic differentiation of BMSCs under CSS, we downregulated NAD^+^ production by siRNA targeting to NAMPT, and upregulated NAD^+^ by supplementing precursor NMN in vitro. Our study found that NAD^+^ acted as an endogenous anti-oxidant and was involved in the regulation of oxidative levels in BMSCs under cyclic stretch. This knowledge brings new perspectives to the knowledge of cell reaction to mechanical environment from the aspect of redox homeostasis. NAMPT, as a key rate-limiting enzyme in the NAD⁺ synthesis pathway, has been widely recognized as an indirect indicator of elevated NAD⁺ levels through its upregulated expression [12, 13]. Although we did not directly measure the levels of NAD⁺ in mandible tissue due to limitations in technical capabilities, the significant upregulation of NAMPT expression provides strong support for the hypothesis of increased NAD⁺ levels (Fig. 3d e). Future studies can further validate this inference by directly measuring NAD⁺ levels. In vivo experiment used precursor NMN and inhibitor FK866 to up or down-regulate NAD^+^ levels. Systemic administration method and administration dosage of NMN was determined according to previous literature [20], which have indicated that systemic administration can achieve stable drug concentrations in the blood. Considering the side effects of FK866 on the systemic hematopoietic system and in order to precisely control the drug concentration in the alveolar bone, we adopted the method of local injection. In our study, we used a 20-g orthodontic force to establish rat OTM models. This force has been shown to induce tooth movement without causing root resorption, in line with the principle of using light forces in orthodontic treatment [41–42]. The osteogenic effect, osteoclastic effect and tooth movement rate in this part of experiment confirmed our hypothesis that NAD^+^ can initiate bone remodeling by promoting the osteogenic and osteoclastic process, ultimately accelerating OTM. Although NMN has not been approved for use worldwide at present, this study confirms that other methods, such as regular sleep patterns, calorie restriction [43], and ketogenic diets [44] to increase NAD^+^, may be a safe and effective way to promote orthodontic tooth movement.

Nrf2 is widely recognized as a key regulator of redox homeostasis that mediates the expression of antioxidant genes through transcriptional regulation, protecting cells from oxidative stress under physiological conditions. In the last decade, it has been found associated to mechanically sensitive tissues [45–48] such as bone tissue and periodontal tissue. Nrf2 causes osteogenesis imperfecta in mice [45]. In this study, we proposed that NAD^+^ preserves oxidative balance in BMSCs through a mechanism involving Nrf2. Regulation of Nrf2 activity and function occurs at transcription, degradation, nuclear translocation, and post-translational modification levels, and etc [49]. qRT-PCR and Western blotting analyses revealed stable Nrf2 transcription and protein expression across si-NAMPT and NC groups in whole-cell samples (Fig. 4A and B). Yet, intra-nuclear protein blot and immunofluorescence indicated impeded Nrf2 nuclear translocation in the NAMPT knockdown group, pointing to NAD^+^’s role in regulating Nrf2 activity through nuclear localization. Nrf2 can be activated by natural compounds [49], drugs [47], and exercise [44], but few studies have investigated its regulatory mechanisms under mechanical stress. Here, we shed new light on the Nrf2 activation mechanism in mechanically stressed BMSCs, suggesting that enhancing NAD^+^ production could be an efficient way to facilitate Nrf2 nuclear translocation. Furthermore, we intend to perpetuate the research with further in-depth discussions on how NAD^+^ influences the nuclear translocation process of Nrf2, thereby elucidating the underlying molecular mechanisms that govern this response to mechanical stress.

**Fig. 7:**
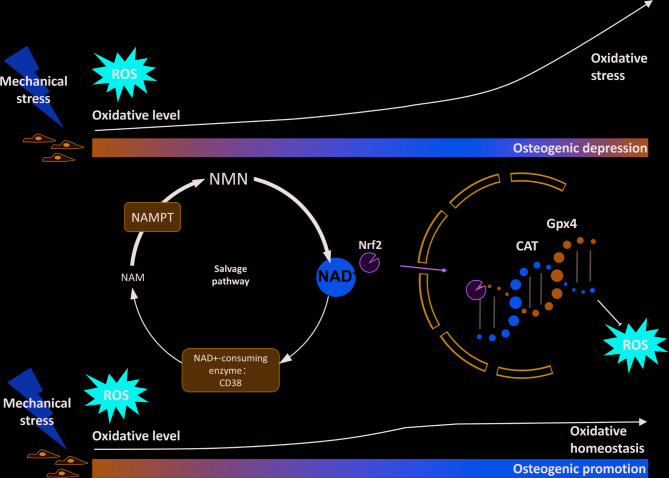
Schematic diagram for the proposed mechanism. BMSCs experience an increase of oxidative level under mechanical stress stimulation. Uncontrolled oxidative products induce oxidative stress and impair osteogenic differentiation. As an endogenous antioxidative metabolite in this process, NAD^+^ formation increase by promotion of NAMPT and impression of CD38. NAD^+^ boosts Nrf2 shuttling to the nucleus, promoting antioxidative gene expression and maintain stable oxidative levels, which ensures the continuous progression of osteogenic differentiation

In summary, our data for the first time directly demonstrate that under mechanical stress, BMSCs maintain intracellular redox balance and prevent cell damage caused by uncontrolled ROS production by regulating Nrf2 and its translocation process through NAD^+^ metabolism (Fig. 7). Therefore, this study may provide new insights into the adaptive regulation of stem cell homeostasis under mechanical stimulation and the acceleration of bone remodeling process.

## Conclusions

In conclusion, our research shows that mechanical stress increases ROS in BMSCs, and the ROS level is modulated by NAD^+^ to protect osteogenic differentiation. We’ve identified the NAD^+^/Nrf2 signaling regulatory pathway, providing insights into BMSC redox balance under mechanical stress. This knowledge could inform molecular strategies for enhancing bone remodeling and other biological processes.

## Electronic supplementary material

Below is the link to the electronic supplementary material.

Additional file 1: Supplementary Table 1. The primer sequences used in this study. Supplementary Table 2. The antibodies used in this study.

Additional file 2: Supplementary Fig. 1 Identification of BMSCs. a The ALP staining of BMSCs after osteogenic induction for 7 days, scale bar = 250 um; Oil red O staining of BMSCs after adipogenic induction for 21 days, scale bar = 500 um; The alizarin red staining of BMSCs after osteogenic induction for 21 days, scale bar = 250 um. b Expression of cell surface markers CD90, CD44, CD45 and CD31 by fow cytometry. Supplementary Fig. 2 Diagram of the in vitro cell tension-loading system. Supplementary Fig. 3 Scheme of experimental OTM model establish and bone morphometric analysis. a The schemes of the rat orthodontic tooth movement and medicine administration experiment in 14-day period. b The intraoral picture of the experimental OTM model. c Quantification of Tb.N of mechanically stimulated models after injection of PBS, NMN, and FK866 on 7 and 14 days. (*N* = 3). d Quantification of Tb.Th of mechanically stimulated models after injection of PBS, NMN, and FK866 on 7 and 14 days. (*N* = 3). e H&E staining of periodontal tissue after injection of PBS, NMN, and FK866 on 7 and 14 days. Scale bar, 100 μm. n.s. *P* ≥ 0.05, Ab, alveolar bone. PDL, periodontal ligament. D, dentin. Supplementary Fig. 4 Immunohistochemistry (IHC) and semi-quantitative analysis of ALP and RUNX2 expression in the bone marrow cavities. a b Immunohistochemistry (IHC) and semi-quantitative analysis of ALP expression in the bone marrow cavities after injection of PBS, NMN, and FK866 on 14 days. Scale bar, 40 μm. c d Immunohistochemistry (IHC) and semi-quantitative analysis of RUNX2 expression in the bone marrow cavities after injection of PBS, NMN, and FK866 on 14 days. Scale bar, 40 μm. e f Immunohistochemistry (IHC) and semi-quantitative analysis of CTSK expression in the bone marrow cavities after injection of PBS, NMN, and FK866 on 14 days. Scale bar, 40 μm. **P ＜0.01, ***P ＜0.001.

Additional file 3: Original images of Western blotting.

## Data Availability

No datasets were generated or analysed during the current study.

## Abbreviations

ALP: Alkaline phosphatase
BMSCs: Bone marrow mesenchymal stem cells
CSS: Cyclic stretch stress
CD38: ADP-ribosyl cyclase 1
CTSK: Cathepsin K
H_2_O_2_: Hydrogen peroxide
NAD^+^: Nicotinamide adenine dinucleotide
NAMPT: Nicotinamide phosphoribosyltransferase
NMN: Nicotinamide mononucleotide
Nrf2: Nuclear factor erythroid 2-related factor 2
OTM: Orthodontic tooth movement
OXPHOS: Oxidative phosphorylation
qRT-PCR: Quantitative real-time polymerase chain reaction
ROS: Reactive oxygen species
