# Supplementary material for: NAD+/Nrf2 signaling promotes osteogenesis by regulating oxidative level of BMSCs under mechanical stress
Source: Prog Orthod. 2025 May 30;26:19. doi: 10.1186/s40510-025-00566-2 (PMC12125440; doi:10.1186/s40510-025-00566-2)
Supplement: Supplementary file 1 — Additional file 1: Supplementary Table 1. The primer sequences used in this study. Supplementary Table 2. The antibodies used in this study. [file 40510_2025_566_MOESM1_ESM.docx]

**Supplementary Table 1.** The primer sequences used in this study.

| Primer Name | Forward Primers (5′–3′) | Reverse Primers (3′–5′) |
| --- | --- | --- |
| *Nampt* | GCCAAGGACTATCTGAACGCTAC | GCAAGTTCCGCCATGATGATTC |
| *Cd38* | GCTGGAGAAGGTGGAGAAGA | CAGGGTGGAGTTGAGGATGT |
| *Runx2* | ACGAATGCACTATCCAGCCA | GCAGGTACGTGTGGTAGTGA |
| *Alpl* | ACAACACCAACGCTCAGGTC | GTGACCTCGTTCCCCTGAGT |
| *Nfe2l2* | CCAGAAGGAACAGGAGAAGGC | GTTTGGGAATGTGGGCAACCT |
| *Gxp4* | GGATGAAAGTCCAGCCCAAGG | CGCAGCCGTTCTTATCAATGAG |
| *Cat* | AGAACATTGCCAACCACCTGAA | GCATTCTTAGGCTTCTGGGAGTT |

**Supplementary Table 2.**The antibodies used in this study.

| Antibodies for western blot | |
| --- | --- |
| NAMPT | Proteintech, NAMPT/PBEF Polyclonal antibody, 11776-1-AP |
| RUNX2 | Cell Signaling Technology, RUNX3/AML2 (D6E2) Rabbit mAb, 9647 |
| ALP | Proteintech, ALPL Polyclonal antibody, 11187-1-AP |
| Nrf2 | Proteintech, NRF2, NFE2L2 Polyclonal antibody, 16396-1-AP |
| CD38 | ABclonal, CD38 Rabbit pAb, A1680 |
| ACTB | Proteintech, beta actin polyclonal antibody, 20536-1-AP |
| Lamin B1 | Proteintech, Lamin B1 Polyclonal antibody, 12987-1-AP |
| Antibody for immunofluorescence staining | |
| NAMPT | Proteintech, NAMPT/PBEF Polyclonal antibody, 11776-1-AP |
| Antibodies for immunohistochemistry | |
| RUNX2 | Cell Signaling Technology, RUNX3/AML2 (D6E2) Rabbit mAb, 9647 |
| ALP | Proteintech, ALPL Polyclonal antibody, 11187-1-AP |
| CTSK | Abcam, ERR24829-101 AB300569 |
