# Supplementary figures and images for: NAD+/Nrf2 signaling promotes osteogenesis by regulating oxidative level of BMSCs under mechanical stress
Source: Prog Orthod. 2025 May 30;26:19. doi: 10.1186/s40510-025-00566-2 (PMC12125440; doi:10.1186/s40510-025-00566-2)

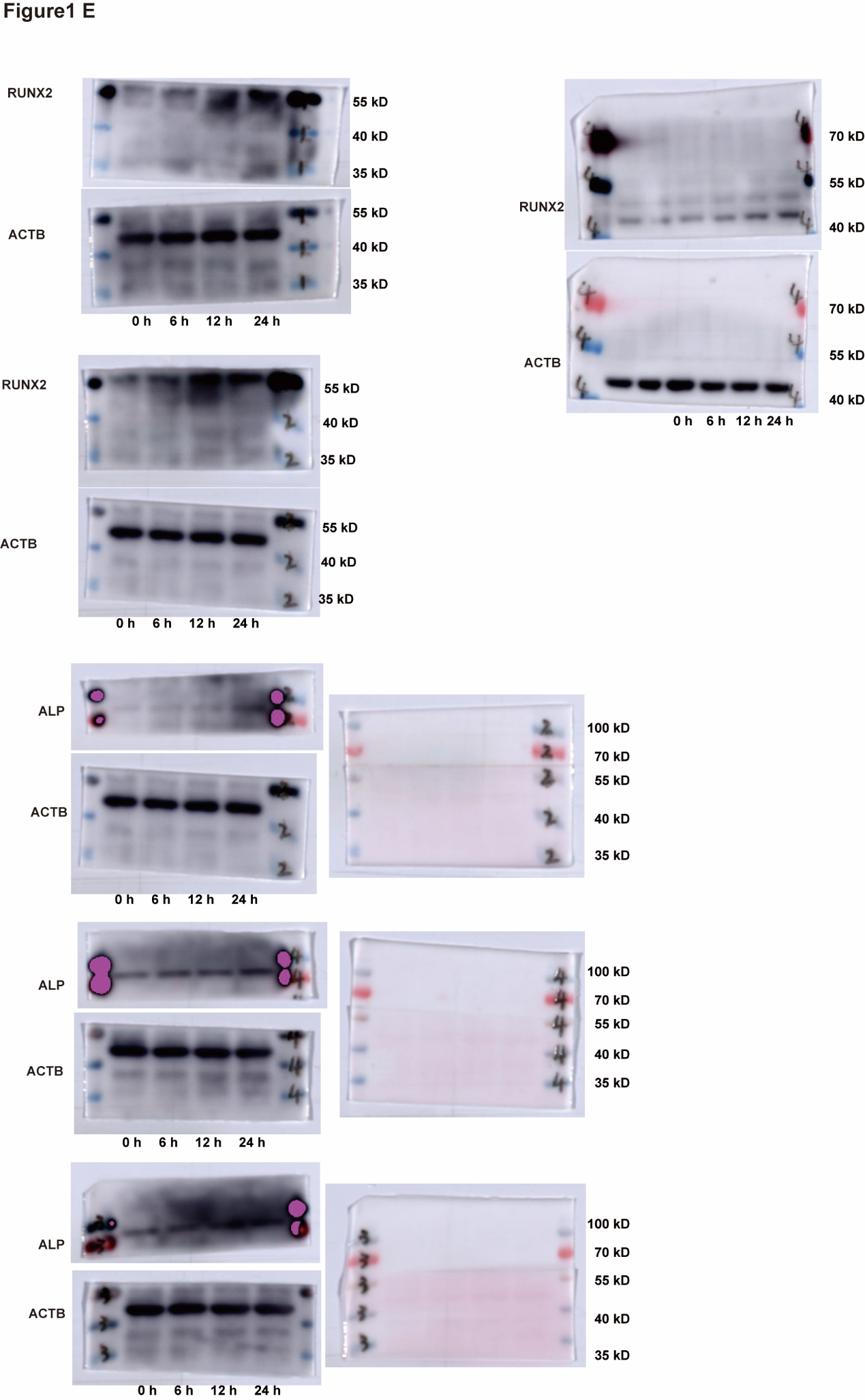


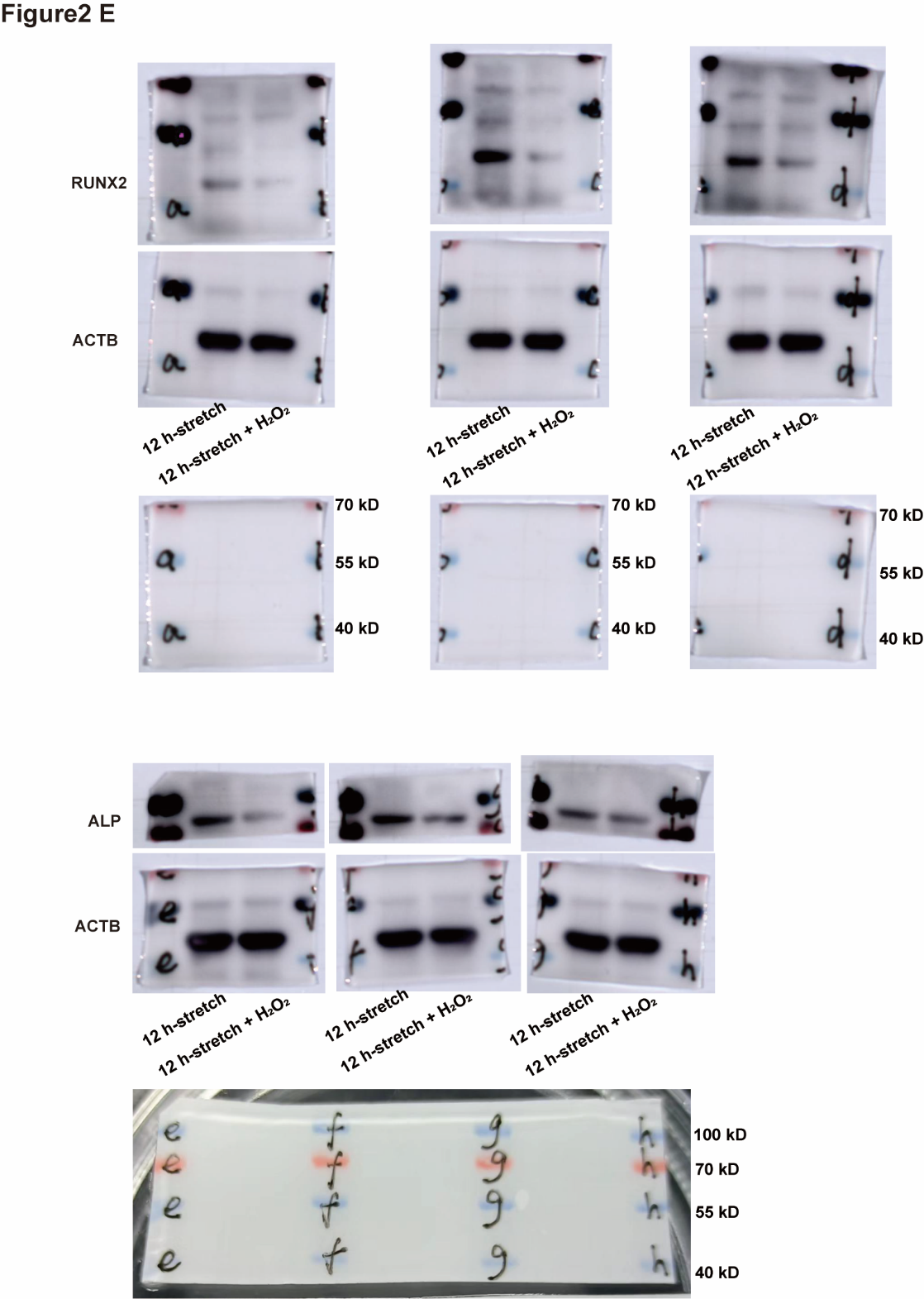


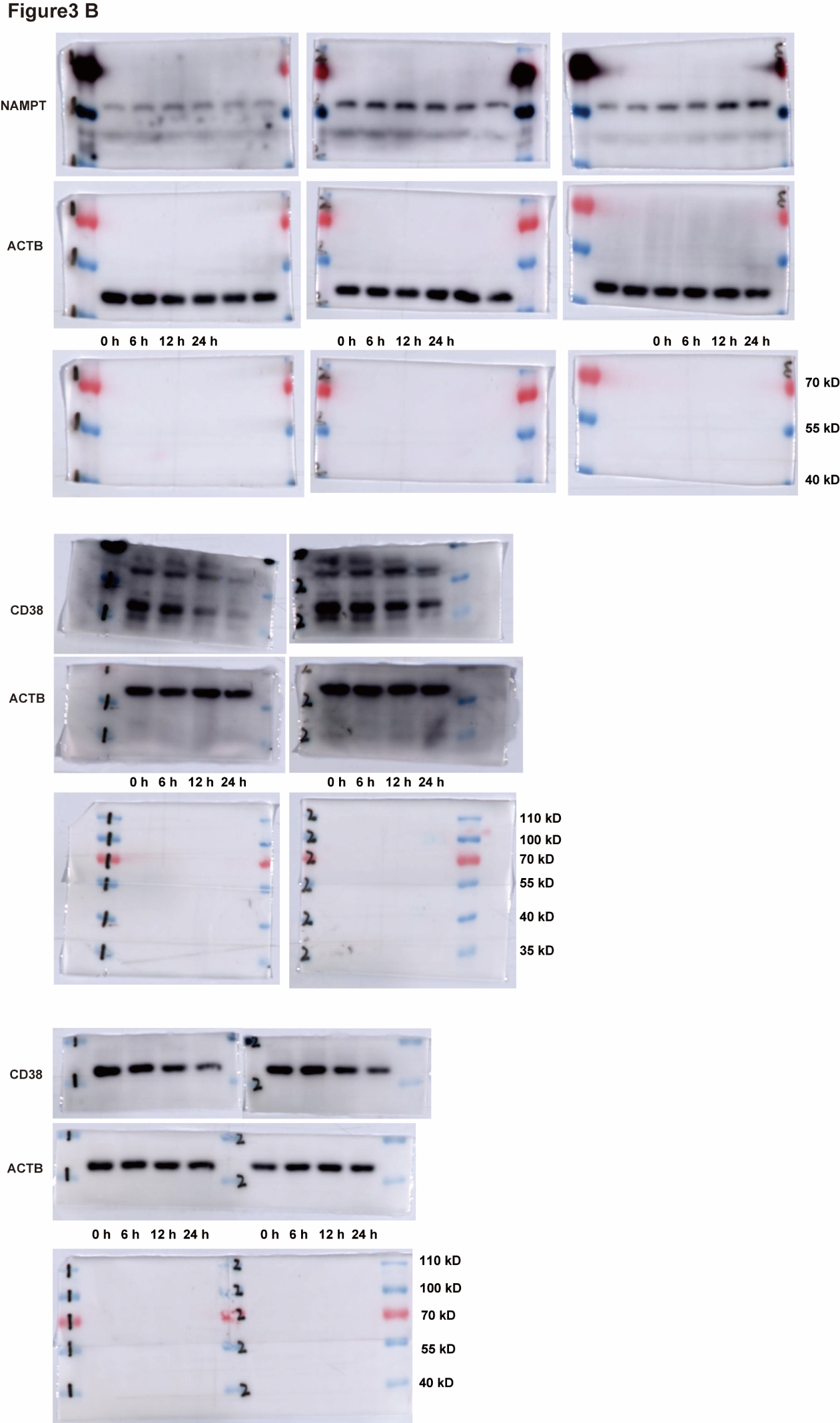


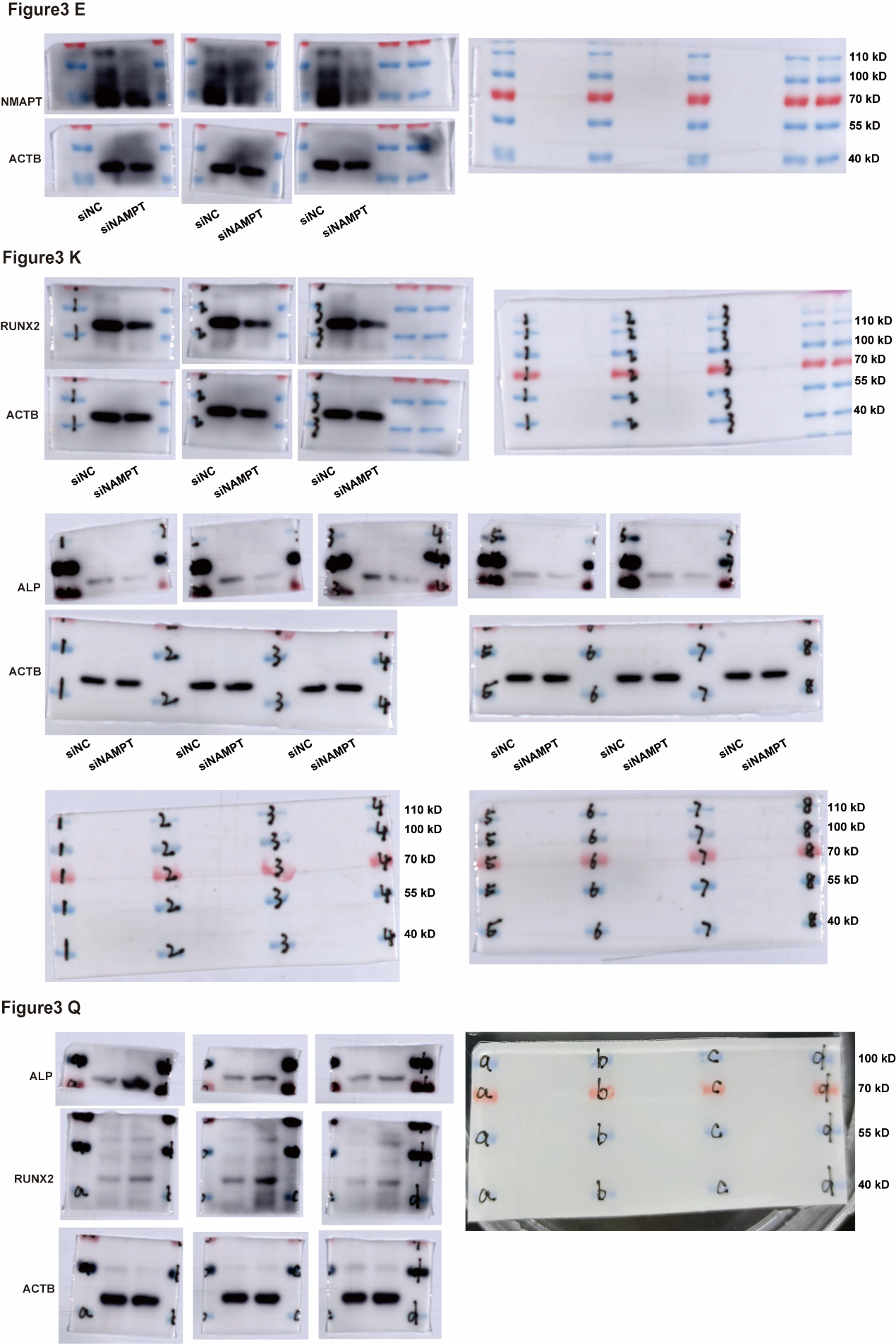


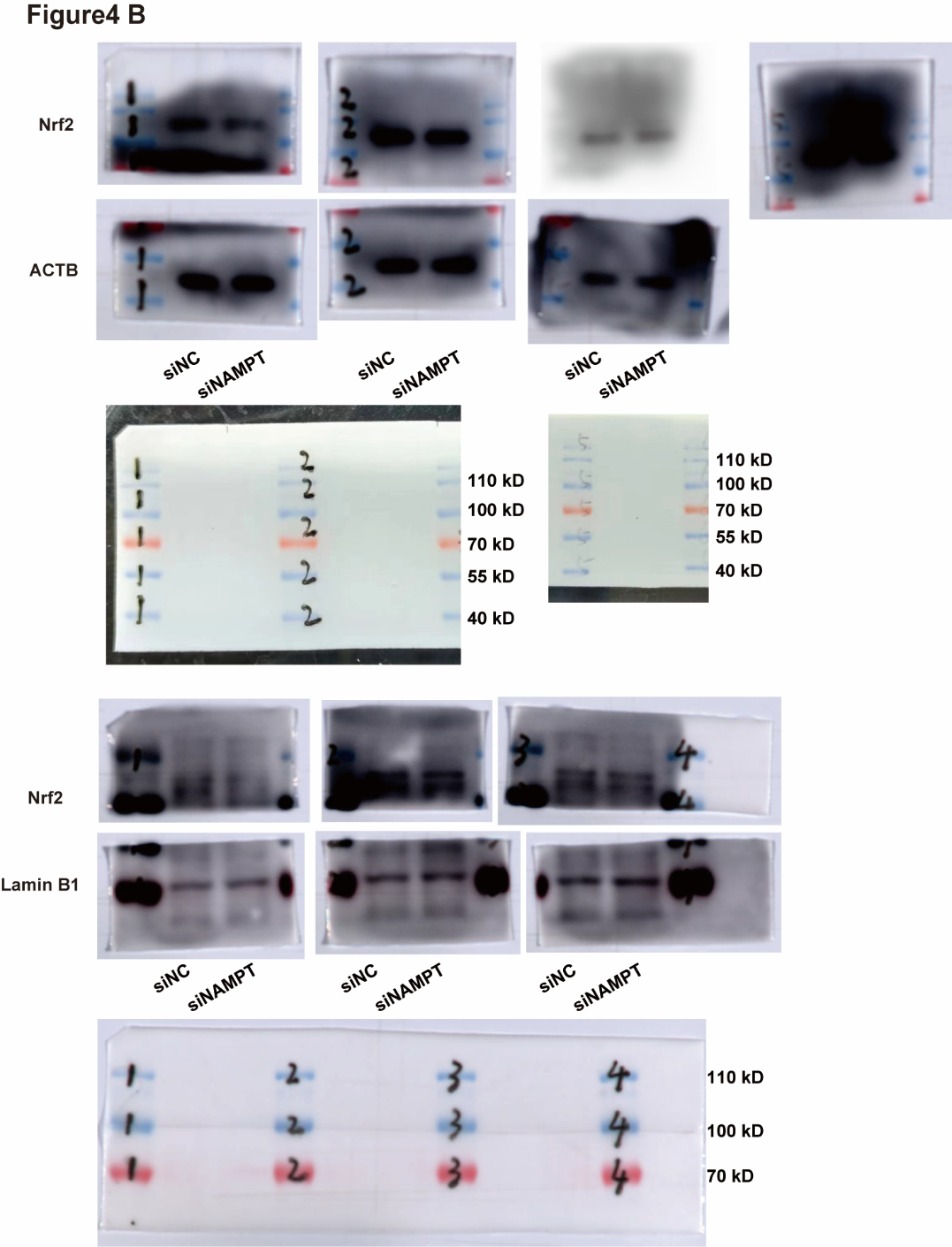


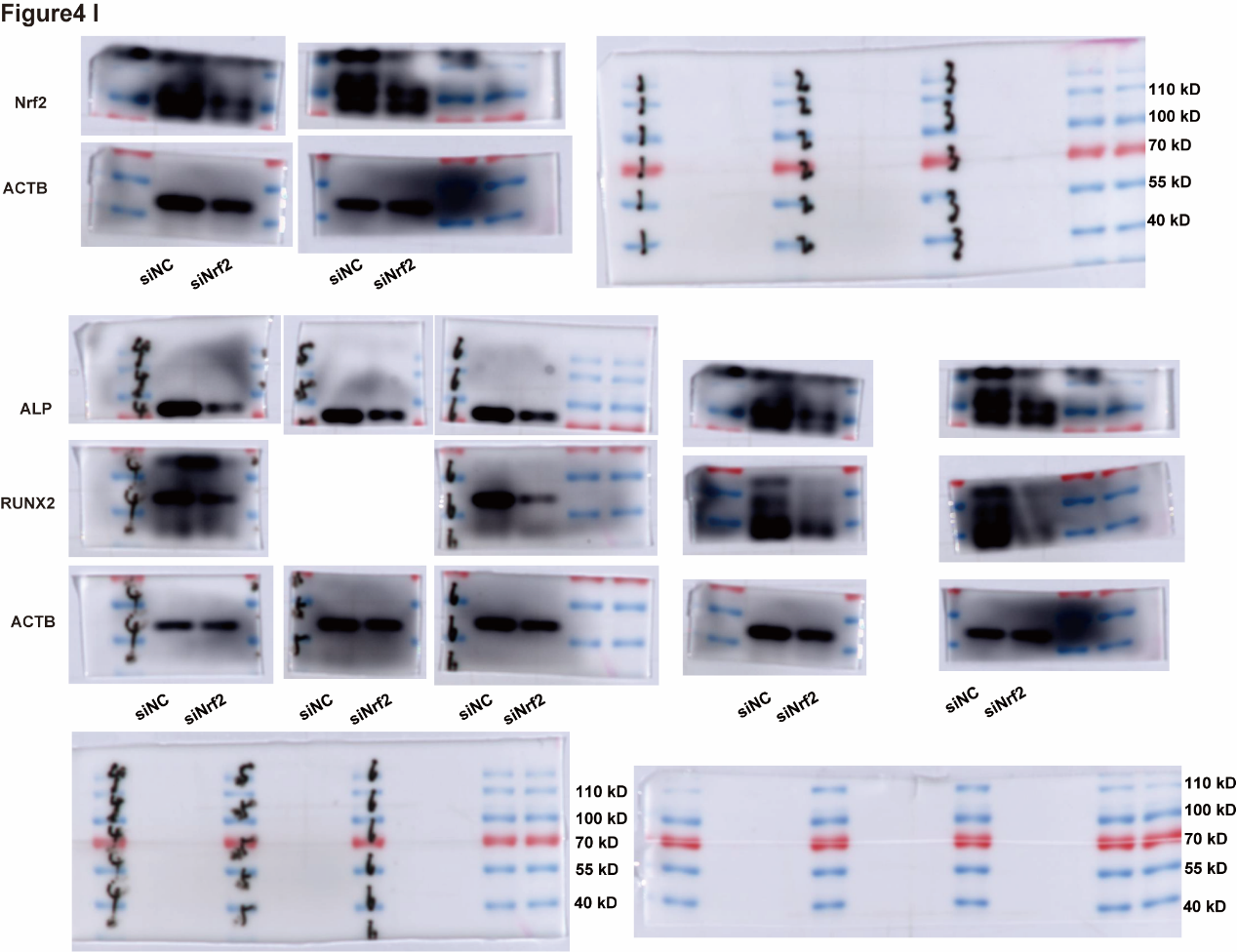

Supplement: Supplementary file 2 — Additional file 2: Supplementary Fig. 1 Identification of BMSCs. a The ALP staining of BMSCs after osteogenic induction for 7 days, scale bar = 250 um; Oil red O staining of BMSCs after adipogenic induction for 21 days, scale bar = 500 um; The alizarin red staining of BMSCs after osteogenic induction for 21 days, scale bar = 250 um. b Expression of cell surface markers CD90, CD44, CD45 and CD31 by fow cytometry. Supplementary Fig. 2 Diagram of the in vitro cell tension-loading system. Supplementary Fig. 3 Scheme of experimental OTM model establish and bone morphometric analysis. a The schemes of the rat orthodontic tooth movement and medicine administration experiment in 14-day period. b The intraoral picture of the experimental OTM model. c Quantification of Tb.N of mechanically stimulated models after injection of PBS, NMN, and FK866 on 7 and 14 days. (N = 3). d Quantification of Tb.Th of mechanically stimulated models after injection of PBS, NMN, and FK866 on 7 and 14 days. (N = 3). e H&E staining of periodontal tissue after injection of PBS, NMN, and FK866 on 7 and 14 days. Scale bar, 100 μm. n.s. P ≥ 0.05, Ab, alveolar bone. PDL, periodontal ligament. D, dentin. Supplementary Fig. 4 Immunohistochemistry (IHC) and semi-quantitative analysis of ALP and RUNX2 expression in the bone marrow cavities. a b Immunohistochemistry (IHC) and semi-quantitative analysis of ALP expression in the bone marrow cavities after injection of PBS, NMN, and FK866 on 14 days. Scale bar, 40 μm. c d Immunohistochemistry (IHC) and semi-quantitative analysis of RUNX2 expression in the bone marrow cavities after injection of PBS, NMN, and FK866 on 14 days. Scale bar, 40 μm. e f Immunohistochemistry (IHC) and semi-quantitative analysis of CTSK expression in the bone marrow cavities after injection of PBS, NMN, and FK866 on 14 days. Scale bar, 40 μm. **P ＜0.01, ***P ＜0.001. [file 40510_2025_566_MOESM2_ESM.docx]
