# Supplementary material for: NAD+/Nrf2 signaling promotes osteogenesis by regulating oxidative level of BMSCs under mechanical stress
Source: Prog Orthod. 2025 May 30;26:19. doi: 10.1186/s40510-025-00566-2 (PMC12125440; doi:10.1186/s40510-025-00566-2)
Supplement: Supplementary file 3 — Additional file 3: Original images of Western blotting. [file 40510_2025_566_MOESM3_ESM.docx]

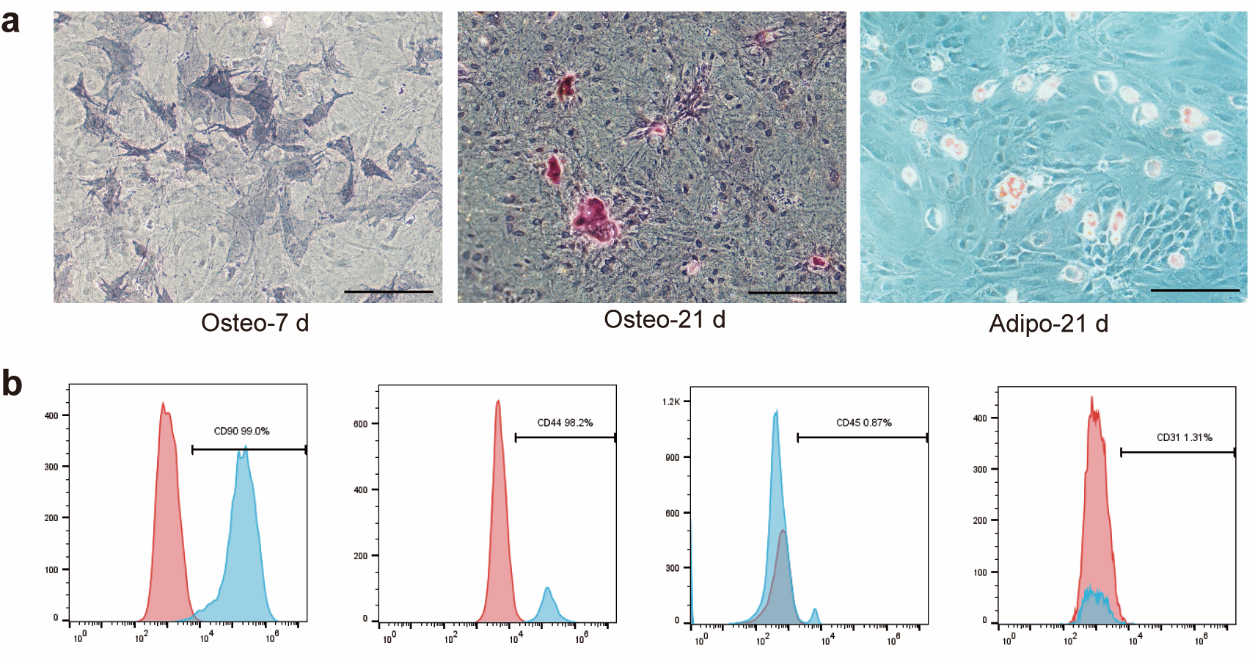


**Supplementary Fig. 1** Identification of BMSCs. **a** The ALP staining of BMSCs after osteogenic induction for 7 days, scale bar = 250 um; Oil red O staining of BMSCs after adipogenic induction for 21 days, scale bar = 500 um; The alizarin red staining of BMSCs after osteogenic induction for 21 days, scale bar = 250 um. **b** Expression of cell surface markers CD90, CD44, CD45 and CD31 by fow cytometry.


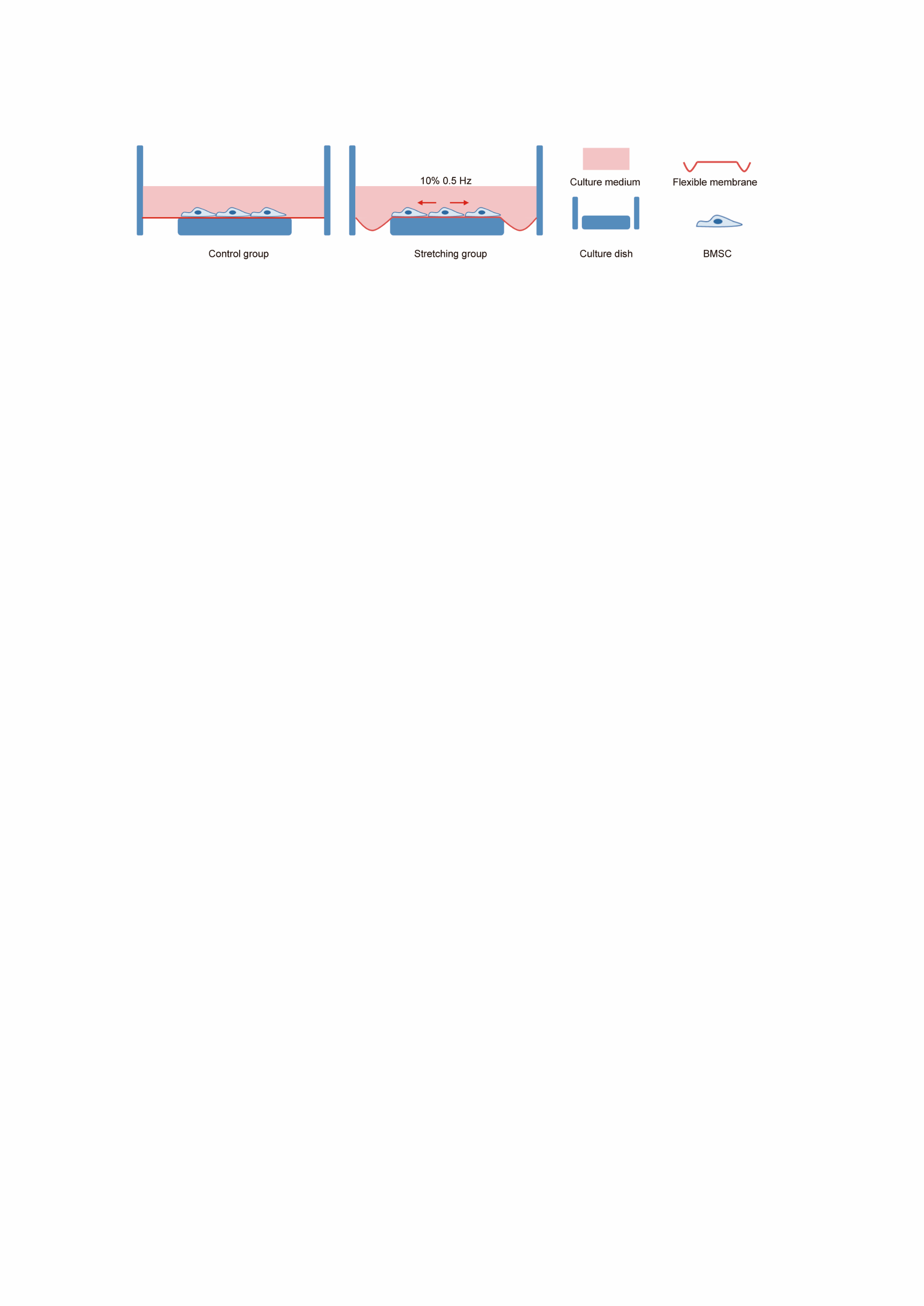


**Supplementary Fig. 2** Diagram of the in vitro cell tension-loading system.


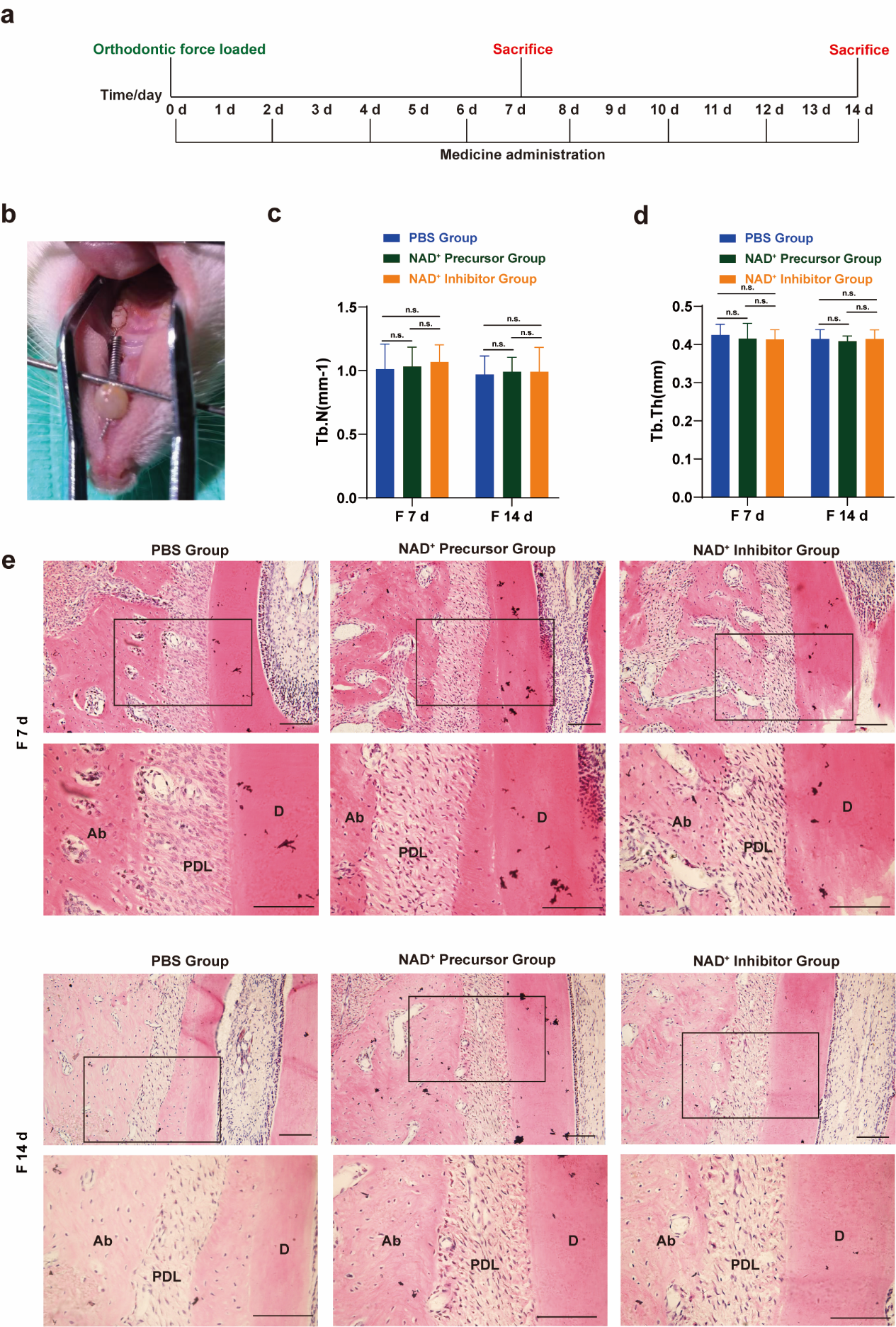


**Supplementary Fig. 3** Scheme of experimental OTM model establish and bone morphometric analysis. **a** The schemes of the rat orthodontic tooth movement and medicine administration experiment in 14-day period. **b** The intraoral picture of the experimental OTM model. **c** Quantification of Tb.N of mechanically stimulated models after injection of PBS, NMN, and FK866 on 7 and 14 days. (*N* = 3). **d** Quantification of Tb.Th of mechanically stimulated models after injection of PBS, NMN, and FK866 on 7 and 14 days. (*N* = 3). **e** H&E staining of periodontal tissue after injection of PBS, NMN, and FK866 on 7 and 14 days. Scale bar, 100 μm. n.s. *P* ≥ 0.05, Ab, alveolar bone. PDL, periodontal ligament. D, dentin.


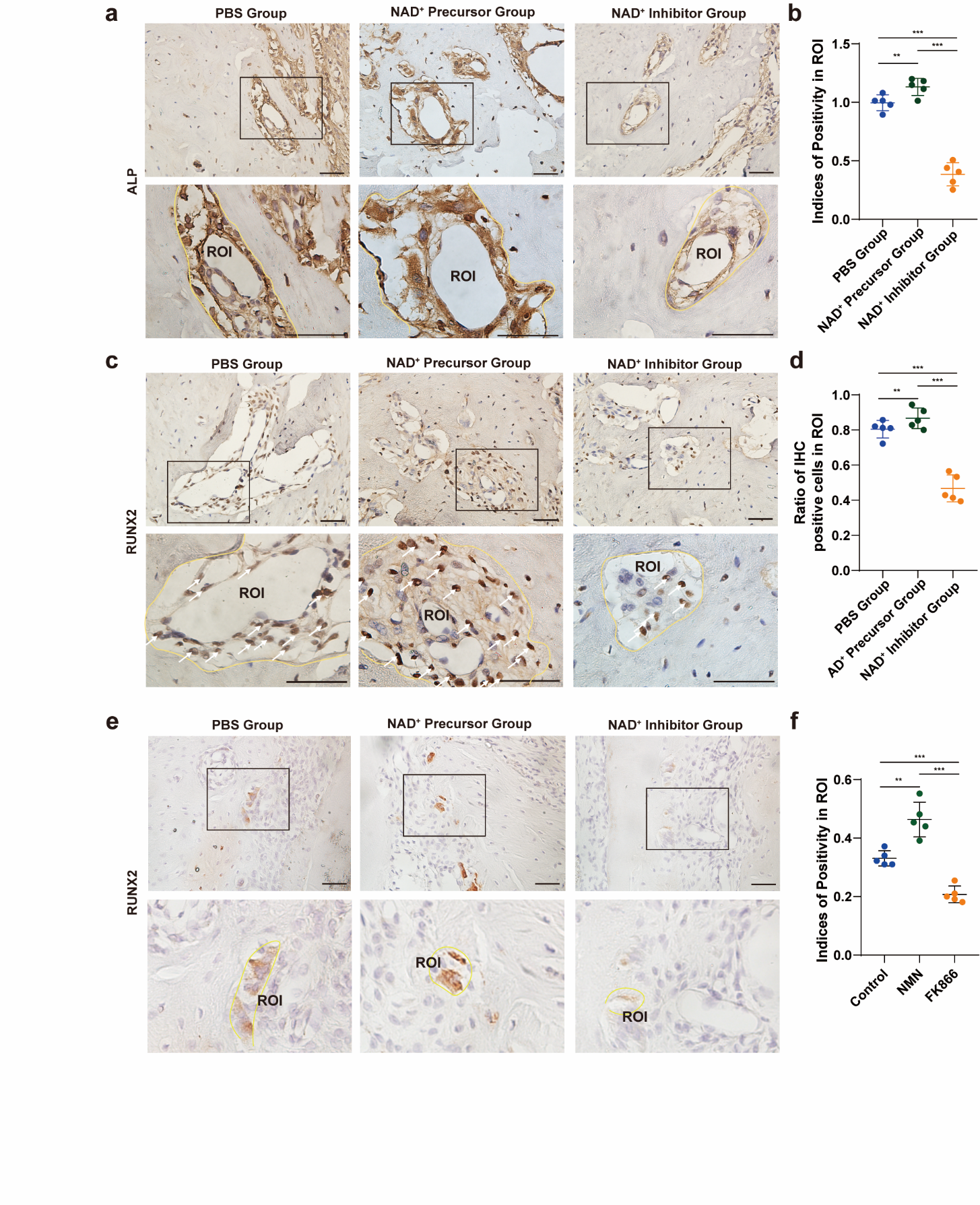


**Supplementary Fig.** 4 Immunohistochemistry (IHC) and semi-quantitative analysis of ALP and RUNX2 expression in the bone marrow cavities. **a b** Immunohistochemistry (IHC) and semi-quantitative analysis of ALP expression in the bone marrow cavities after injection of PBS, NMN, and FK866 on 14 days. Scale bar, 40 μm. **c d** Immunohistochemistry (IHC) and semi-quantitative analysis of RUNX2 expression in the bone marrow cavities after injection of PBS, NMN, and FK866 on 14 days. Scale bar, 40 μm. **e f** Immunohistochemistry (IHC) and semi-quantitative analysis of CTSK expression in the bone marrow cavities after injection of PBS, NMN, and FK866 on 14 days. Scale bar, 40 μm. ** *P*＜0.01, *** *P*＜0.001.
